# Various Aspects of Antimicrobial Properties of *Populus* Propolis

**DOI:** 10.3390/ijms27219718

**Published:** 2026-10-29

**Authors:** Piotr Okińczyc, Jarosław Widelski, Jakub Szperlik, Zbigniew Sroka

**Affiliations:** 1 Department of Pharmacognosy and Herbal Medicines, Wroclaw Medical University, 211A Borowska Street, 50-556 Wrocław, Poland; zbigniew.sroka@umw.edu.pl; 2 Department of Pharmacognosy with Medicinal Plant Garden, Medical University in Lublin, 1 Chodźki Street, 20-093 Lublin, Poland; jaroslaw.widelski@umlub.edu.pl; 3 Laboratory of Tissue Culture, Botanical Garden, Faculty of Biological Sciences, University of Wrocław, 23 Sienkiewicza Street, 50-234 Wrocław, Poland; jakub.szperlik@uwr.edu.pl

**Keywords:** propolis, poplar, *Populus*, antibacterial, review

## Abstract

Propolis is a bee product which is composed of plant resins and bee glue. In temperate climatic zones, the main sources of resins are buds of different species of the *Populus* genus. Propolis is known to have multiple medicinal properties, especially antimicrobial (including antibiotic-resistant bacterial strains), anti-inflammatory, and wound treatment properties. Due to growing antibiotic resistance, new antibacterial agents and therapies solutions are still needed. For this reason, this article summarizes the results of research related to the antibacterial activity of *Populus* genus propolis. We discuss the main types of *Populus* genus propolis (aspen, aspen–poplar and poplar), propolis’s general effect against different bacterial strains, the classes of antibacterial agents, the mechanism of antibacterial action of propolis, and interactions of propolis with antibiotics. Our analysis of the literature reveals that *Populus* propolis is a valuable antibacterial agent. However, its activity varies due to the instability of its composition. Therefore, before it can be designated for medicinal use biological and phytochemical standardization is required. Clinical data reveal the efficiency of propolis preparations in mild external infectious diseases in oral cavity and wound treatment. These data are limited only to poplar propolis. Similar efficiency may be possible using aspen and aspen–poplar propolis, but there is no direct clinical data to support this assertion. Combined therapy using propolis with antibiotics remained controversial due to the lack of sufficient clinical data and unclear data from in vitro research. Administering *Populus* propolis preparations to treat internal bacterial diseases requires further clinical research and should not be a standard therapy at this moment.

## 1. Introduction

Propolis, also called bee glue, is one of the most important products of honeybees (*Apis mellifera* L.). It is a mixture of plant secretions and resins which are mixed with beeswax and a small admixture of other components. A typical propolis coming from the temperate climate zones consists of about 20–50% balsam/resinous fraction, 30–70% beeswax, and 4–10% volatile compounds (they are part of the resin fraction). Other components of propolis are pollen (about 5%) and mechanical impurities, which represent less than 5% [1,2].

To date, numerous scientific studies have reported on the medicinal properties of bee glue. It is effective against microorganisms such as fungi, viruses, and protozoa. In addition to the mentioned activity, propolis also exhibits antioxidant, antiradical, anti-inflammatory, anticancer, apoptosis-stimulating, wound healing and analgesic activity [3,4,5,6,7]. All these properties are widely used in folk medicine and in academic medicine to a limited extent. These limitations of propolis usage are caused by the instability of its chemical composition. Different factors are responsible for this. However, the most important factors encompass differences in local flora around world, bee preferences for using specific resins, many beekeeping techniques and the variability of weather during the vegetation season [8]. As a result, the instability of propolis’s composition strongly limits its applicability to external and mild disorders. However, specific standardized preparations of propolis may find wider application. Another way to take advantage of propolis’s beneficial properties is to utilize the most active propolis fraction as a natural matrix for the development of future multicomponent drugs. Special attention is given to the activity of propolis extracts against bacteria [3,4,9], which are increasingly resistant to the most popular antibiotics—a serious problem in the treatment of bacterial infections. This option requires accurate investigations of propolis’s chemical composition, searching for active substances and investigating their impact on the whole of propolis’s activity as well as analyzing the interactions between propolis components.

There are many reviews of propolis. However, most of them analyze general medicinal properties and/or propolis composition, and antibacterial activity is only partially analyzed. Moreover, review articles focused on propolis’s antibacterial activity usually describe mainly poplar propolis types (or propolis from different species origins) without distinguishing further between *Populus* species propolis subtypes. Therefore, the present review focuses on the main types of propolis originated from *Populus species* and their antibacterial activity. The scope of the review includes classifying *Populus* propolis subtypes and the differences between their chemical compositions, comparing their antibacterial activity, describing their main antibacterial agents and mechanisms of action, discussing the interactions between propolis and antibiotics, and the problems of standardizing propolis and its use in practical medicine.

## 2. Criteria of Review Article Selection

The documents used to write the review were divided into two categories: “basic literature data” and “supported literature data”. Basic literature data was mainly found in the Scopus^®^ and Pubmed^®^ databases. The keywords “propolis” and “antibacterial” were used for the preliminary selection of documents. Scopus^®^ contained 1692 documents, while Pubmed^®^ listed 970 on 16 October 2026. From these records, articles about *Populus* propolis and its antibacterial activity were directly selected. *Populus* propolis is the type of propolis of which the plant precursors are different species of the *Populus* genus. The main criterion used to select articles about *Populus* genus propolis was the composition of samples and the presence of specific marker components. (Marker components are further described in Section 2). If the composition of investigated samples was unavailable, especially in older articles, the selection criterion was the samples’ origin. If only geographical origin was available, and it was in agreement with the spread of poplar propolis plant precursors (especially *P*. *nigra*), it was assumed that the investigated samples had poplar origins, but only for in vitro research. In the case of clinical trials, samples without a defined composition were categorized as being of unknown plant origins. Apart from articles found in the basic databases, we also included theses and books written by propolis specialists. As a result, our “basic literature data” was composed of 82 research articles (74 in vitro research, three in vivo models and five clinical trials), ten review articles, two theses, and five books.

**Figure 1:**
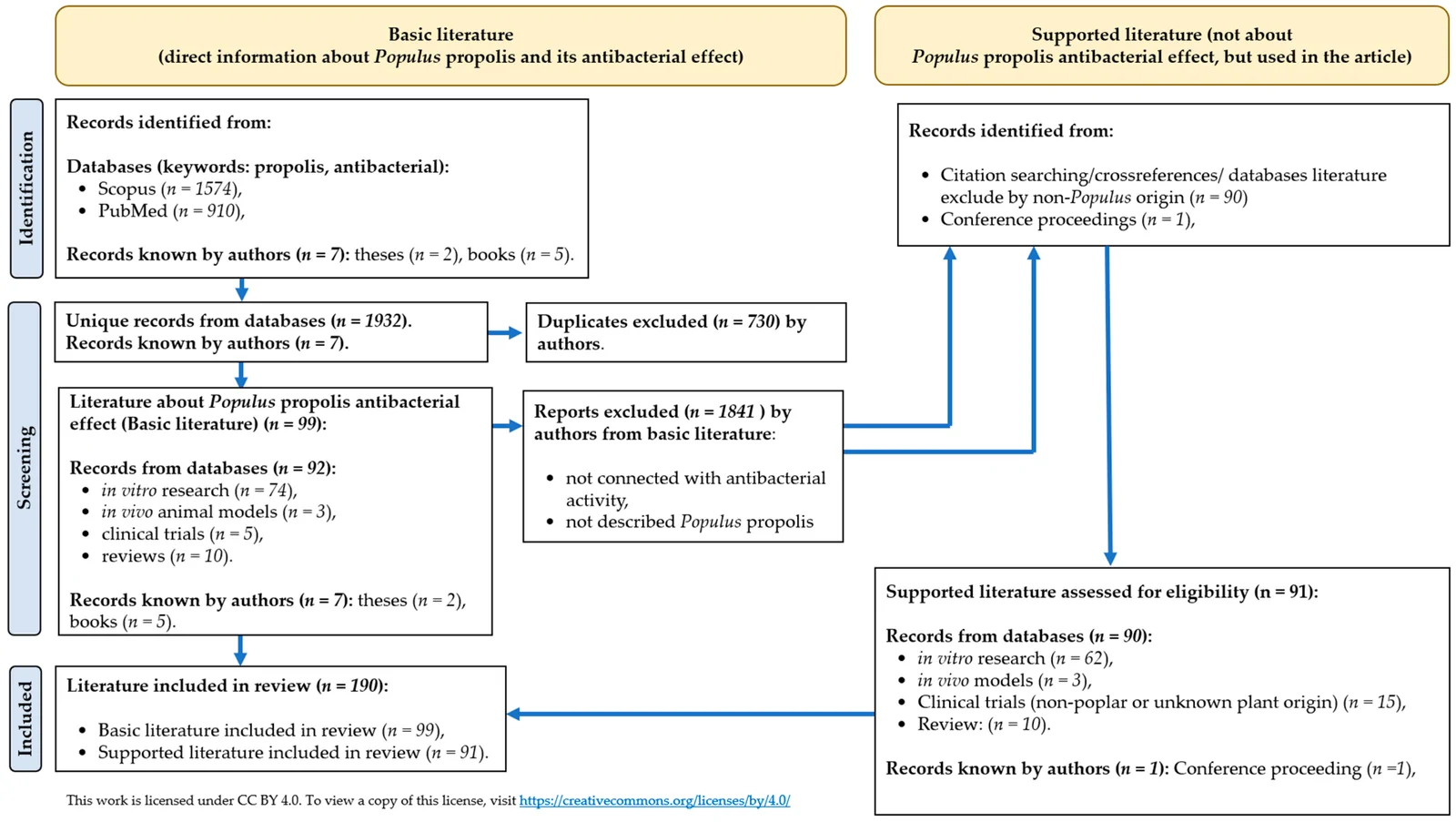
Prisma graph of literature review.

The “supporting literature data” consists of records found by citation search/citation searching/cross-references. This literature contains research articles 80 (62 in vitro research, three in vivo models and fifteen clinical trials), ten review articles and one conference proceeding. It was used to supplement basic information about Populus propolis in every section of the article. Our literature analysis is presented as a PRISMA graph in Figure 1. Graph was prepared due to work of Page et al. [10].

### Classification of Propolis, Type of Propolis Originated from Populus Resins and Other Propolis Types

The main components of propolis are beeswax and plant resins and exudates. The differences of propolis composition depend on the plant resins (exudates) used by bees. The type of a resin’s plant source (propolis plant precursor) is determined by investigation of the propolis’s composition and a search for markers specific to certain plant precursors [6,11]. These investigations are usually based on LC-UV/VIS-MS [12,13,14,15] or GC-MS [6,16,17,18] research. During analysis, the type of propolis is determined by the presence and and concentration of specific marker component peaks in chromatograms and comparisons with literature. If propolis contains mainly markers characteristic (usually defined as the highest/most abundant/strongest peaks on chromatograms) of only one plant precursor, it is classified as a “pure” type. In this case, markers for another propolis type should be absent or present as trace components—as very small peaks in comparison to dominant components. Usually, the trace components must be below than 1/10–1/20 of the area of the peaks of the main components, according our personal expertise. However, different researchers use their own criteria. If there are no distinctly dominant markers, the propolis is classified as mixed [6,16,19]. Apart from simple classification, several statistical methods were also used for more accurate classification. The most common methods included hierarchical fuzzy clustering (dendrograms) [6,16,20] or principal components analysis (PCA) [13,21,22,23,24,25]. Both techniques are based on an analyses and transformation matrix which is built from different described concentrations of components in propolis’s phytochemical profile or specific physiochemical parameters of analyzed samples. The amounts of singular components are described in the mass of substances [15,25], percentage of GC-MS [6,16] or LC-UV [13,21,22] peaks or direct amounts of specific markers’ ions [24,26]. All of these specific parameters are compared with exudates from *Populus* species buds, either directly or from results from literature. Contrary to simple classification based on peaks dominance, these complex techniques are more reliable and allow us to distinguish between mixed propolis samples (or more reliably describe them if a classified sample is mixed using mathematically defined criteria). These methods allow to distinguish several specific types of *Populus* propolis. This was possible due to the strong preferences of bees for collecting resins from one species [6,11]. For this reason, it is suggested that usually propolis has one main dominant plant precursor [6,11]. In temperate climates (Europe, North America, non-tropical regions of Asia, South America, New Zealand), bees prefer collecting poplar secretions (*Populus* spp.) [6,11,13].

Historically, previous investigations were most often based on poplar propolis originating from black poplar trees (*P*. *nigra*). Therefore, this type of propolis was called poplar or black poplar propolis. However, newer investigations have provided similar phytochemical profiles of black poplar and other *Populus* species, especially *P*. *balsamifera* (syn. *P*. *tacamahaca*) [27]. Moreover, *P*. *nigra* and *P*. *balsamifera* are known for their easy creation of crossbreed species [27]. Further research revealed more species with similar composition to *P*. *nigra*, such as *P*. *suaveolens* and *P. simonii* [12]. For this reason, the poplar propolis type describes samples of propolis of similar phytochemical composition to black poplar. The main (strong) markers of this propolis type are caffeic acid monoesters (1,1-dimethylallyl ester and their geometric isomers and phenylethyl ester-CAPE) [2,6,13,16,28] as well as flavonoids aglycones (chrysin, tectochrysin, galangin, pinocembrin, pinostrobin, and pinobanksin and their derivatives—pinobanksin-3-acetate and other 3-esters and pinobanksin-5-methyl ether) [2,6,13,16,28]. However, not all propolis which belong to poplar-type always contain all of the mentioned flavonoids and caffeic acids monoesters. These are the results of differences of composition between different *Populus* species. For example, there are known chemotypes of *P. nigra* which do not contain one of the most basic poplar propolis markers—pinobanksin-3-acetate and pinobanksin-5-methyl ether—but do contain high amounts of hydroxycinnamic monoesters, especially CAPE and 1,1-dimethylallyl ester and their geometric isomers [12].

The second propolis type which originates from *Populus* sp. resins is the aspen propolis type. Its main precursors are aspen poplars (*P*. *tremula*, *P*. *tremuloides*), grand leaf Asian poplars (*P*. *lasiocarpa*, *P*. *wilsocarpa*) [28] and African poplar—*P. euphratica* [29,30]. Distinct from poplar propolis, the aspen type contains lower amounts of flavonoids and has its own specific marker—hydroxycinnamic acids glycerides. Some exudates of *P. tremula* contain mainly mono-hydroxycinnamic acid and di-hydroxycinnamic glycerides. In some derivatives, glycerol is additionally esterified by one or two acetic acid molecules [13,22,31]. Some components from this group also known as lasiocarpins, as they were isolated from *P. lasiocarpa* by Asakawa et al. [32]. Among them, the main markers of aspen propolis are 1,3-di-*p*-coumaroylglycerol, 2-acetyl-1,3-di-*p*-coumaroylglycerol (lasiocarpin A), 2-acetyl-1-*p*-coumaroyl-3-feruloylglycerol and 2-acetyl-1,3-diferuloylglycerol [6,15,16,27,28].

**Figure 2:**
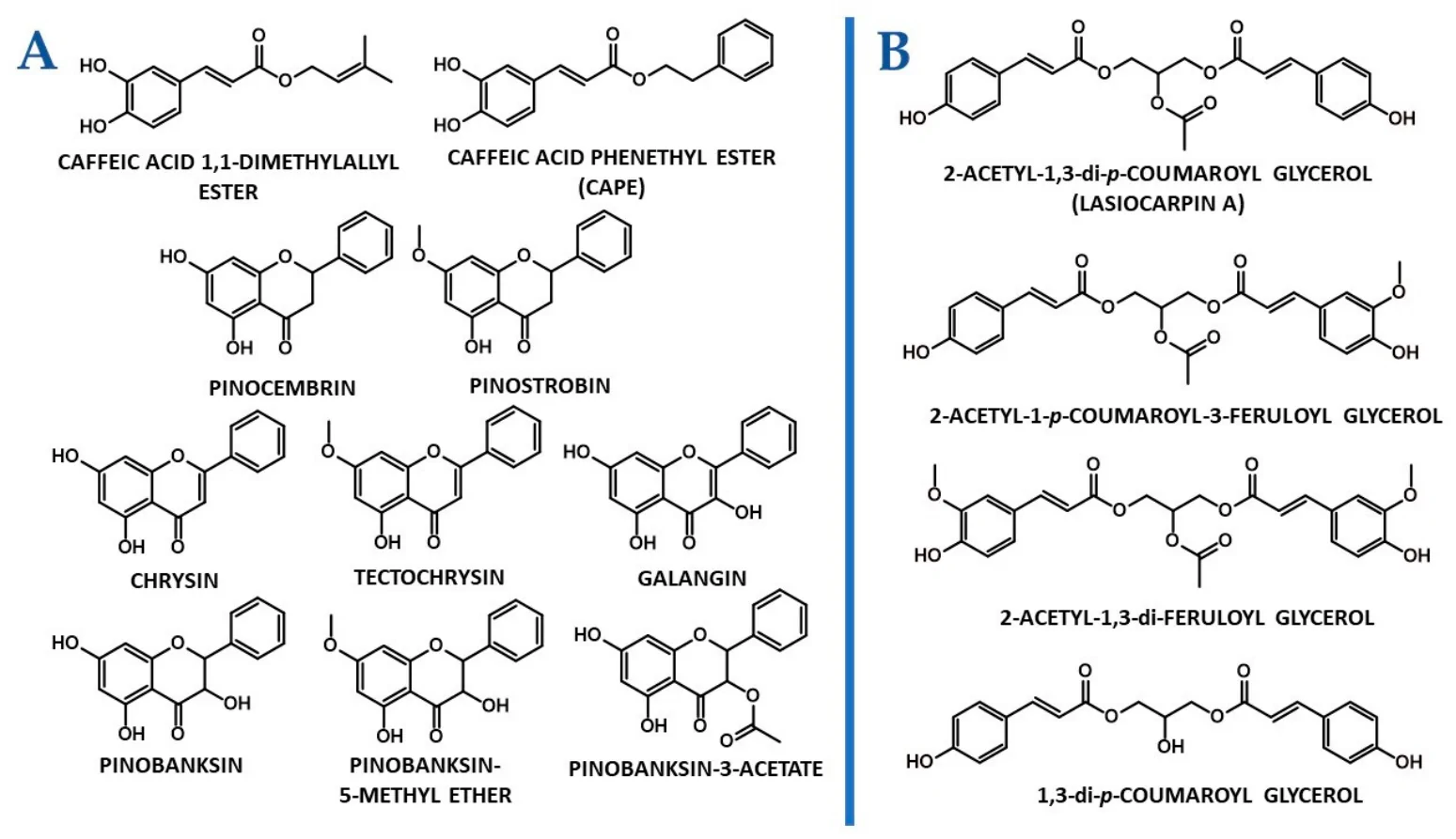
Strong markers of aspen (**A**) and poplar (**B**) propolis type.

The last type of *Populus* genus type propolis is aspen–poplar propolis. This type may be a mix of resins from poplar propolis (ex. *P*. *nigra*) and aspen propolis (ex. *P*. *tremula*) or it may have only one plant precursor. It has been determined that the resins of some *Populus* species, especially *P. szechuanica* (syn. *P*. *violascens*), contain components characteristic of *P. tremula* (2-acetyl-1,3-dicoumaroylglycerol 1,3-dicoumaroylglycerol, 1-coumaroyl-3-caffeoylglycerol, 2-acetyl-1-caffeoyl-3-coumaroyl-glycerol) and *P. nigra* (pinocembrin, pinocembrin chalcone, pinostrobin chalcone, chrysin, pinobanksin, pinobanksin-3-acetate, CAPE, etc.) [16]. The main markers characteristic of poplar and aspen propolis were presented in Table 1 and Figure 2 at the end of this section.

In practice, it is impossible to accurately track propolis’s origin without a wide investigation of local poplar flora. This phenomenon is caused by several factors:

- *Populus* species are known for their easy formation of crossbreed specimens that are difficult to distinguish from “pure” species. This situation is complicated by the historical introduction of many foreign poplar species in many areas. (Some poplar trees are ornamental plants as well as sources of biomass.) [27,33,34].
- Bees are known for being selective when seeking material to make propolis, and sometimes they may ignore the trees closest to their hive, especially if the trees are foreign species [35].
- Bees are also opportunistic organisms, and if the most desirable material is absent or in too low supply for the bees’ purposes, they use other resins or add them to the primary material [6,17,36].

Even though poplars are one of the most common trees, they are not natural flora in all areas. It is possible that in some local flora, the frequency of individual trees is too low to meet the needs of bees. Therefore, bees are forced to seek alternative raw materials. In cold regions of Russia, they collect the secretions of birch trees (*Betula* spp.) [6,11], while in tropical regions the main plant precursors are *Baccharis dracunculifolia* DC. [11,37], *Dalbergia* species [38,39], *Macaranga tanarius* (L.) Müll.Arg. [40,41] *Clusia* species [42,43] etc. A comparison table of the main described propolis types (with one main precursor) is presented in Table 1 below. This table includes only strong markers for specific propolis types. However, there are also components which are present in different types of propolis, but in different amounts. Their concentration (or the proportions between them) may be used as a secondary marker of propolis’s origin, but it should not be treated as a decisive factor. For *Populus* propolis (distinguishing between poplar and aspen type), these components are free hydroxycinnamic acids (caffeic, *p*-coumaric, ferulic and isoferulic), flavonoid aglycones (sakuranetin, naringin) and *p*-coumaric acid benzyl ester [13,16,22,28].

Apart from these pure non-poplar kinds of propolis, there are also known types which contain resins of the *Populus* genus and another non-*Populus* genera. These types occur when an area of poplar distribution overlaps with the distribution areas of non-poplar resinous plant genera (for example aspen–poplar–birch type [17]). Moreover, the introduction of *Populus* species to new areas causes observations of black poplar genus markers in unexpected areas such as Chile [44]. Due to the different chemical compositions of typical *Populus* genus propolis and strongly limited data, all these mixed types are not part of the present review.

In summary, every propolis research project should accurately analyze its chemical composition to avoid misinterpretation of the propolis’s plant precursors. Moreover, further conclusions about the activity of propolis from a specific origin requires the analysis of as wide a range of samples as possible to ensure sufficient reproducibility. Accurate phytochemical analysis is important, because it shows differences in propolis’s qualitative and quantitative composition, even within the same area [13,45]. In the case of mixed samples, even analyses of minor components (and minor plant precursors) may be required to draw the right conclusions, because the final activity of propolis depends on interactions between different components.

**Table 1:** Comparison of main propolis types with one dominant plant precursor.

Propolis Type	Plant Precursor	Strong Marker Components	Geographical Spread	Rf
Poplar	Balsamic poplars: *Populus nigra* L., *P. balsamifera* L. etc.	Hydroxycinnamic acids non-glyceride monoesters, specific flavonoid aglycones,	Temperate climate hemisphere and poplars plant zones	[6,11,13]
Aspen	*Populus tremula* L., *P. tremuloides*, *P. lasiocarpa*, *P. wilsocarpa* etc.	Hydroxycinnamic acids glycerides	Temperate climate hemisphere	[11,13]
Birch	*Betula* spp.	Sequiterpenoids, some specific flavonoids	Colder region of Russia	[6,16]
Mediterranean	*Cupressaceae* family	Diteterpenes	Mediterranean area	[46,47,48]
Green	*Baccharis dracunculifolia* DC.	Prenlylated phenolic acid derivates	Tropical areas (Brazil, Nepal, China)	[11,37]
Red	*Dalbergia* spp.	Neoflavonoids, isoflavonoids, pterocarpans	Tropical areas (Brazil, Nepal, China)	[38,39]
Clusia	*Clussia* spp.	Benzophenones	Cuba, South America	[42,43]
Pacific	*Macaranga tanarius*	C-prenylated flavonoid aglycones	Pacific region; Indonesia, Japan (Okinawa)	[40,41]

## 3. General Antibacterial Effect of Populus Propolis, Resistant and Susceptible Bacterial Strains and Comparison of Activity with Plant Precursors

Despite the fact that propolis around the world has variable qualitative and quantitative compositions, it exhibits a similar profile of biological activity. Differences are usually observed in the level of antibacterial activity rather than in the profile itself [3,7,9,11,49,50]. Antibacterial effects are described as bactericidal [51,52,53], bacteriostatic [51], and species [52] and dose-dependent [52]. Mirzoeva et al. [52] claimed that propolis works in two stages. In the initial stage, very active components of propolis act and the effect is bactericidal. This stage is short due to the easy hydrolysis of antibacterial component. During the second stage, weaker but more stable components act, and the observed effects in this longer period are bacteriostatic. As a result, the effects of whole extracts may be bactericidal if the effect of labile strong components (their concentration) outclasses the effect of weaker components. Moreover, the authors did not observe any adaptation of bacterial strains to propolis [52].

Among Gram-positive strains, activity was most often tested against *Staphylococcus*, *Streptococcus*, *Bacillus*, and *Enterococcus* species. The tested Gram-negative bacteria usually included *Escherichia coli*, *K. pneumoniae*, *Pseudomonas aeruginosa*, and *Helicobacter pylorii.* These research revealed that typical *Populus* genus propolis has stronger activity against Gram-positive bacteria than Gram-negative ones (except for some species, especially *Helicobacter pylori* [21,54,55,56,57,58]) and fungi [2,9,50,57]. However, some researchers have found levels of activity (only a two-fold difference of MIC in some cases) against typically propolis-resistant Gram-negative (*Escherichia coli*, *Pseudomonas aeruginosa*) similar to the activity observed against propolis-susceptible Gram-positive (*Staphylococcus aureus*, *S. epidermidis*) bacterial strains [59].

Most of the antibacterial research of *Populus* species propolis to date has been performed on ethanolic extracts, usually 70% ethanol in water extracts (the typical abbreviation is 70EEP or sometimes EEP) or pure ethanolic extracts (the typical abbreviation is EEP). Basically, this solution originated from traditional and folk medicine [52] and uses a relatively low toxicity ethanol preparation which allows it to be directly used. From a practical point of view, the biologically active components which are part of the propolis’s balsamic fraction are the most important [2,7,49]. The remaining propolis fractions, especially beeswax, are ballast material. Therefore, the basic preparation of propolis relies on the separation of the balsamic fraction from beeswax and other ballast material. Due to the apolar nature of beeswax, it exhibits worse solubility in semi-polar organic solutions than the balsamic fraction. For these reasons, the best solution for the separation of beeswax and the rest of the ballast material from the balsamic fraction is to extract the propolis in a semi-polar solution such as 70% ethanol in water. Moreover, comparison research of pure ethanolic and different ethanolic-water extracts showed that the strongest antibacterial agents are extracted at about 60–80% of ethanol concentration [60]. A similar phenomenon was also observed for non-poplar propolis types [61,62,63], which may be evidence for the specific selection of plant resins by bees.

Antibacterial investigations of propolis have been performed using different methods, including disc diffusion tests [7,22,50], microdilution methods [7,17,42,44,52,55,58,59,60,61,62,63], time-kill assays [52,64], microscopic research [65,66] and antibiofilm assays [64]. The majority of research was performed using microdilution methods, and they are used in this article for comparison purposes (Table 1). A comparison of the antibacterial potential of different *Populus* propolis extracts against different bacterial strains is presented in Table 2. It is based on minimal inhibitory concentration values (MIC) according to the widest range of research papers.

Comparison of the MIC values of *Populus* propolis showed that most of the performed research was focused on poplar propolis types. Data about aspen and poplar-aspen type is strongly limited. This is probably because of the stronger presence of poplar propolis in the market. Moreover, much of the available research either does not contain any data about the composition of the investigated samples, or only partially investigated the propolis’s phytochemical profile. For these reasons, plant precursors may be only suspected due to the propolis’s geographical origin, and the addition of aspen or phytochemically similar species resins cannot be entirely excluded in some cases. However, some of the available research includes accurately defined phytochemical profiles of propolis samples as well as the plant precursors. These investigations show that the 70EEP of poplar propolis should be a rather stronger antibacterial agent than 70EEP from aspen–poplar and aspen types [21,72]. However, Isidorov et al. [6] discovered that diethyl ether extracts of aspen buds had stronger antibacterial activity than extracts from black poplar buds. Vardar-Ünlü found similar activity in a methanol extract of *P*. *nigra* and *P*. *tremula* buds and poplar propolis [51]. Further research revealed that the composition of *P. nigra* [6,12,13,16,22] and *P. tremula* [6,13,16,22] bud extracts are variable, which may impact on their antibacterial activity. In the case of poplar propolis free of aspen-derived material, the composition is dominated by three groups of compounds: free hydroxycinnamic acids (caffeic, *p*-coumaric and ferulic acids), monoesters of hydroxycinnamic acids (primarily non-glycerol monoesters of caffeic acid), and flavonoid aglycones [6,12,13,16,22]. Consequently, these constituents exhibit varying levels of antibacterial activity, and “pure” poplar propolis itself may demonstrate stronger or weaker antibacterial effects depending on its specific composition.

In summary, it could be said that *Populus* propolis is a strong antibacterial agent and its activity is often potent against even drug-resistant bacterial strains. It may be hypothesized that the poplar propolis type (especially 70EEP) should be a rather stronger antibacterial agent than aspen and aspen–poplar type, but not in all cases. The relationships between *Populus* propolis’s antibacterial properties, its composition, and its mechanism of action are further described in next paragraph. A significant limitation of the comparisons presented in this chapter is the incomplete knowledge—or even total lack of information—regarding the composition of the propolis samples in many studies. Consequently, it is not always possible to identify the botanical precursors of these samples. Furthermore, different researchers employed varying extraction and solvent evaporation procedures, which can influence the activity of the final product. Due to the influence of these numerous unknown factors on activity, the results presented in this section provide an approximate indication of antibacterial action rather than a definitive determination of which type of *Populus* propolis exhibits the strongest antibacterial properties.

## 4. Antibacterial Agents of Populus Propolis

### 4.1. Connection of Different Components with Antibacterial Activity

The most important aspect of *Populus* species propolis is the lack of direct correlation of antibacterial activity with any one specific component. Research often reveals correlations with more than one component [7]. Moreover, it is a known fact that lone propolis components had lower antibacterial activity than the most active fraction of propolis and sometimes whole dried/lyophylized extracts [5,7,50]. All these observations suggest that the whole antibacterial effect of *Populus* species propolis is a result of interactions between different components. Propolis EOs have their own antibacterial effect [8,9], and combinations of some non-propolis EOs with dried EEP [86] and 70EEP [87] exhibited synergistic effects against different bacterial strains. All of this data suggests that propolis EOs should have impacts on antibacterial activity. However, to our best knowledge, researchers did not investigate their direct impact on the antibacterial activity of dried/lyophylized propolis extracts, which are standard in antibacterial research on propolis. Although EO components are common substances in propolis liquid extracts, most of them are removed from extracts during solvent evaporation [88]. The complete removal of solvents from previous extracts is required, because many of them, especially ethanol, are strong antibacterial agents and their presence has led to disruptions of antibacterial research. For these reasons, research on EOs in dried/lyophilized extract has a lower priority for scientists than research of non-volatile components.

In the case of non-volatile components, researchers found correlations between strong antibacterial effects and entire groups of components (total amount of polyphenols [2,45,55,89,90], flavonoids [55,89,90], flavones and flavonols [45], flavanones and dihydroflavonols [45]) as well as singular components, especially specific flavonoids aglycones [21,55,91] and hydroxycinnamic acids non-glycerol monoesters [7,21]. However, there is not always a clear correlation between a whole group of components and/or singular components observed. Most often, correlations with flavonoids content [7] and the total amount of polyphenols [45,64] are not observed. This is probably caused by the varied compositions of the investigated propolis and different methodologies used in research.

In summary, the concentrations of specific flavonoid aglycones and hydroxycinnamic acids non-glycerol monoesters are important to the antibacterial activity of *Populus* propolis, but the status of EOs is unclear. All these classes of components are further described in the next paragraphs.

### 4.2. Flavonoids

The flavonoids present in *Populus* propolis are mainly aglycones and their esters with organic acids. Glycosides are almost absent in *Populus*-type propolis (only apigetrin, apigenin-7-glucoside was observed both in pure poplar propolis [28,72] and *Populus* resin [12]). Typical flavonoid aglycones for poplar propolis are flavans (pinocembrin, pinostrobin, sakuranetin, isosakuranetin), flavons (chrysin, tectochrysin), flavan-3-ol (pinobanksin), flavon-3-ols (galangin, quercetin, kaempherol) and chalcones (pinostrobin chalcone, pinocembrin chalcone and pinocembrin dihydrochalcone) [2,6,13,16,28,68,72]. In case of flavonoid esters, this group contains only esters of pinobanksin (estrificated in position 3-OH). Accurate comparison of all these components’ antibacterial activity is limited due to the lack of data, especially direct comparisons of antibacterial activity using this same methodology.

**Figure 3:**
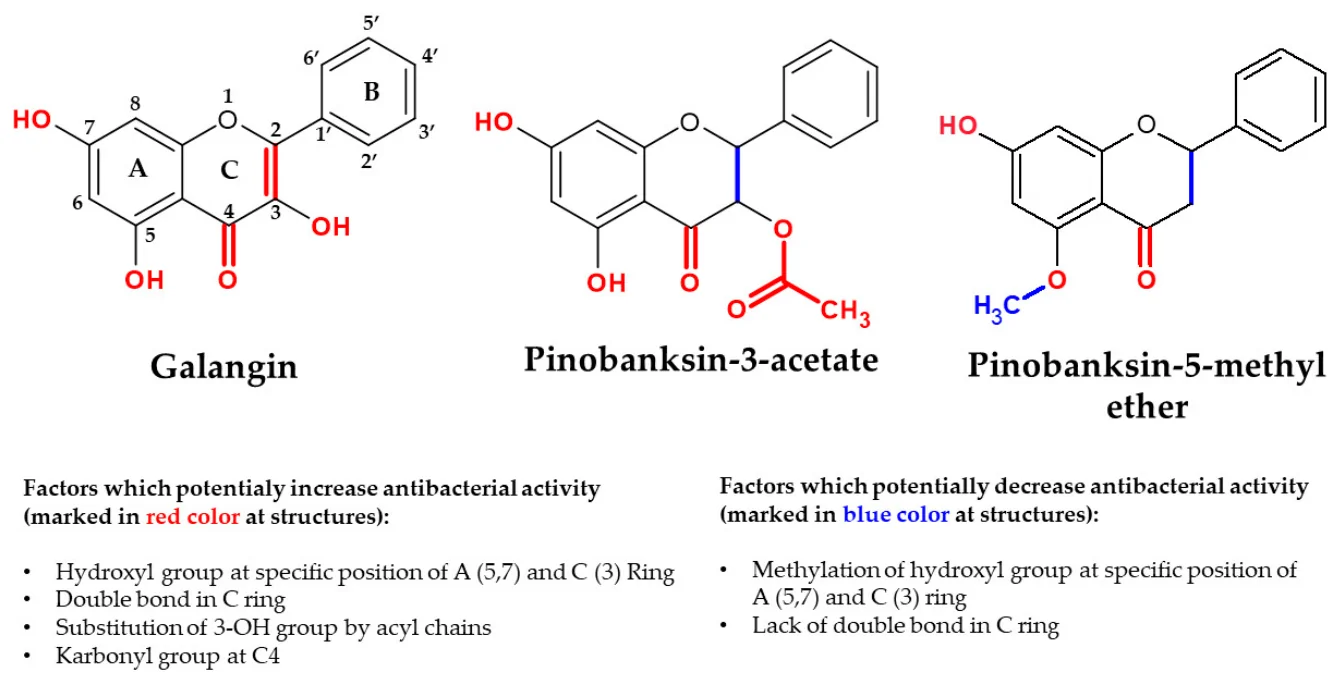
Structural relationship between poplar propolis flavonoids and their antibacterial potential and flavonoid numeration (see galangin).

The research on the relationship of the antibacterial action of flavonoids and their structure demonstrated that this activity is determined by a number of factors, such as the presence of a carbonyl group in the C4 position, the substitution of a hydroxyl group in a specific position of the flavonoid structure and their substitution (or lack of substitution), the presence of a double bond in position 3 and an open C ring (or closed) [92,93,94]. Among these factors, the most important is the presence of a carbonyl group at C4, OH groups at position C5, C7 ring A, C3 in a C ring and a lack of these groups in the B ring [92,93,94]. Moreover, a substitution of the 3-OH group by acyl chains as well as the presence of a double bond in position C3 also increase antibacterial activity [94]. A chalcone structure may also be crucial for activity against some bacteria [92,93,94]. Some flavonoids associated with antibacterial activity fulfill described rules, especially galangin and pinobanksin esters, but not all of them (see Figure 3). Theoretically, methylation of an OH group at the C5 or C7 position should lead to lower antibacterial activity [92,93,94], but some research [21,55] suggested that the presence of pinobanksin-5-methyl ether may be important for activity against *Helicobacter pylori*. One potential explanation for this observation may be an unknown interaction between potentially weaker and stronger propolis antibacterial agents, which lead to increased activity against specific pathogens. These interactions may occur between flavonoid and non-flavonoid classes of components.

High concentrations of most these components relate to strong antibacterial activity in poplar propolis and aspen–poplar propolis. Research has revealed correlations between chrysin [91], pinocembrin [91], galangin [7,22,91], pinobanksin-3-acetate [91], apigenin [89], kaempferol [89], tectochrysin [89] and pinocembrin-7-metylether (pinostrobin) [89] and the antibacterial activity of poplar propolis extracts against *S. aureus*. Similar flavonoid aglycones were also correlated with activity against other Gram-positive bacteria such as *Enterococcus faecalis* (chrysin [21,91], pinocembrin [91], galangin [21,91]), *S. epidermidis* (chrysin [21]), *B. subtilis* (galangin [22], pinostrobine chalcone [22]) and *M. luteus* (chrysin [21]).

Gram-negative bacteria are usually more resistant to *Populus* propolis, but some research has found correlations between antibacterial effects and flavonoids aglycones such as pinocembrin chalcone (vs *E. coli* [22]). In the case of the susceptible Gram-negative bacteria *Helicobacter pylori,* flavonoids aglycones (pinobanksin-5-methyl ether [55], chrysin [55], pinocembrin [55], galangin [55], pinobanksin-3-acetate [55], pinobanksin-3-butanoate or isobutanoate [55] and pinostrobin [55]) and phenolic acid esters (methoxycinnamic acid cinnamyl ester [55]) were correlated with a strong antibacterial effect.

Due to the complex composition of propolis, researchers have also investigated potential interactions between different flavonoid combinations. Kharsany et al. [95] found a synergistic effect against *L*. *monocytogenes* (pinocembrin-galangin, galangin-chrysin) and *P*. *aeruginosa* (galangin-chrysin). They also noted additive effects against *S*. *aureus*, *E*. *faecalis*, *E. coli* and *L*. *monocytogenes* of different combinations of chrysin, galangin, and pinocembrin. Moreover, no antagonistic effects were found. In previous research, Kaleta [7] determined that the right amounts and proportions of pinocembrin, chrysin, galangin, and pinobanksin-3-acetate in artificial combinations are crucial for antibacterial activity against *S. aureus*. However, the most active fractions isolated from poplar propolis EEPs had relatively lower amounts of these components than less active fractions. Moreover, the MICs of these fractions were 18-34 µg/mL, while anyone who tested the components (caffeic, p-coumaric, and ferulic acids as well as chrysin, pinocembrin, galangin and pinobanksin-3-acetate) did not report MICs *S. aureus* in concentrations of 50 µg/mL.

In summary, flavonoids have an important role in propolis antibacterial activity, but the activity of whole extracts is not a function of the same flavonoid concentrations.

### 4.3. Hydroxycinnamic Acids and Their Non-Glycerol Monoesters

Hydroxycinnamic acids and their non-glycerol esters are groups of components which include caffeic, *p*-coumaric, ferulic and isoferulic acids and their derivatives (monoesters). Among free hydroxycinnamic acids, either *p*-coumaric acid or caffeic acid is usually present in *Populus* propolis in the highest concentration [16,28,72,96]. Lower amounts are usually observed for ferulic and isoferulic acid [16,28,72,96]. Generally, free hydroxycinnamic acids are components of weaker antibacterial activity than specific propolis flavonoids, and hydroxycinnamic acids esters and their artificial mixtures are inactive in the same concentration as propolis EEP [7]. In the context of whole propolis antibacterial activity, usually researchers found a lack of impact on propolis antibacterial activity [7] or even negative impacts [7,21,22,55]. The observed negative impact was probably caused by statistical comparisons of samples strong dominated by free hydroxycinnamic acid, with poplar propolis rich in flavonoid and hydroxycinnamic acid monoesters. Moreover, artificial mixtures of the same phenolic acids, such as EEP, (at similar concentrations to those found in propolis extracts) did not exhibit antibacterial activity in this same concentration [7]. This suggests their marginal impact on whole poplar propolis extracts’ antibacterial activity if it contains a higher concentration of another polyphenols class, especially flavonoids and hydroxycinnamic acids non-glycerol monoesters.

A stronger impact on the antibacterial activity of poplar propolis was exhibited by monoesters of hydroxycinnamic acids. That the impact on the antibacterial activity of hydroxycinnamic acid non-glycerol monoesters is stronger than that of free hydroxycinnamic acids relates to the esterification of the carboxyl group, increasing its lipophilicity and allowing for easier penetration of bacterial cell barriers. A similar phenomenon was observed for propolis flavonoids. Research showed that pinobanksin has lower antibacterial activity than pinobanksin-3-acetate [97]. The main non-glycerol monoesters of propolis are derivatives of caffeic acid (phenethyl ester-CAPE, benzyl ester-CABE, 1,1-dimethylallyl ester and its geometric isomers, usually described as prenyl and isoprenyl esters), *p*-coumaric acid (benzyl and 1,1-dimethylallyl ester and its geometric isomers) and methoxycinnamic acid cinnamyl ester. Different researchers observed varying impacts on antibacterial activity among these components. In the case of CAPE, some researchers found a positive impact on propolis antibacterial activity [7] against *S. aureus*, while another showed a lack of impact [22]. Moreover, investigations of artificial combinations of flavonoid aglycones and phenolic acid non-glycerol monoesters suggested that they have a weaker impact on whole antibacterial activity (against *S. aureus*) than flavonoid aglycones [7]. Further research showed different interactions between hydroxycinnamic acids monoesters and flavonoids. For example, CAPE used in combination with eriodyctyol or quercetin exhibited antagonism, but its combination with luteolin showed synergism [98]. Therefore, it may be concluded that interactions between flavonoids and hydroxycinnamic acids monoesters are complex and defy simple prediction. Apart from CAPE, there is also information about connections between geometric isomers of caffeic 1,1-dimethylallyl esters and the antibacterial activity of propolis. These components exhibited stronger activity against *S. aureus* [99] than typical propolis flavonoids (chrysin, galangin, pinocembrin, and pinobanksin-3-acetate). Moreover, Kartal et al. [100] tested the antibacterial potential of isomeric mixtures of 3,3-dimethylallyl caffeate and isopent-3-enyl caffeate, and it demonstrated a stronger antibacterial effect than previous propolis extracts against several Gram-positive bacterial strains (*S. aureus*, *S. epidermidis*, *E. faecalis*, *B. subtilis* and *Corynebacterium diphtheriae*). On the other hand, another research project did not find a direct correlation of caffeic acid prenyl ester and its geometric isomers with the antibacterial effect of whole extract [7,21,22,55]. A similar situation was noted for caffeic acid benzyl esters. However, indirect data (activity against *Paenibacillus larvae*, a pathogen causing American foulbrood in bees [101]) suggested its role in the antimicrobial activity of whole poplar propolis extracts. In the case of *p*-coumaric acid esters, it is suggested that some of them, such as *p*-coumaric acid cinnamyl ester, may play an important role in antibacterial activity against *B. subtillis* and *P. aeruginosa* [22]. A lack of direct correlation for other *p*-coumaric acid esters may be results of their lower proportions in comparison to other components. It can be concluded that the role of hydroxycinnamic non-glycerol monoesters in whole extracts is complex and it is not a simple function of their concentration.

### 4.4. Hydroxycinnamic Acids Glycerides

The last group of hydroxycinnamic acids derivatives in propolis are hydroxycinnamic acids glycerides. Some aspen and aspen–poplar propolises contain mainly acetyl-di-hydroxycinnamic acids glycerides, while non-acetylated and mono-hydroxycinnamic acids glycerides are described in lower amounts [6,13,16,22]. The main hydroxycinnamic acids in the esterified glycerol molecule are *p*-coumaric, caffeic and ferulic acids [6,13,16,22].

Distinct from non-glycerol hydroxycinnamic acid monoesters, the status of hydroxycinnamic acids glycerides’ impact on antibacterial activity is not so clear. To our best knowledge, there are no direct data about the antibacterial activity of singular hydroxycinnamic acid monoesters. Some indirect data suggest that they may be weaker antibacterial agents than flavonoid aglycones and hydroxycinnamic monoesters [7,21,22,55]. Due to this data, the presence of hydroxycinnamic acids glycerides in propolis is usually connected with lower expected antibacterial activity. However, it was also showed that an extract from *P. tremula* sometimes exhibited stronger effects than *P. nigra* extracts [6,21,51]. This may suggest some antagonistic reaction between hydroxycinnamic acids glycerides and some polyphenolic components of *P. nigra* exudates. However, this hypothesis requires further research.

### 4.5. Volatile and Semi-Volatile Components

Comparative research has found that essential oils (EOs) of propolis have their own antibacterial activity, sometimes even stronger than that of 70% propolis extract [9]. According to investigations, poplar propolis may consist of as much as 10% volatile components [1,2]. Many of them are known for their antibacterial effects, and for these reasons they may play a role in the whole antibacterial effect of propolis. These components included monoterpenes and monoterpenoids (e.g., pinene, geraniol), sesquiterpenes and sesquiterpenoids (e.g., α and *β*-eudesmol, caryophyllene, δ-cadinene), simple phenols (vanillin, eugenol), benzoic acid and its esters (benzyl benzoate, benzyl salicylate and benzyl cinnamate) and some wax alcohols [8,13,22,102,103]. The typical EO of *Populus* propolis is usually a mix of different sesquiterpenoids and esters of aromatic acids, with monoterpenes and simple phenols at lower concentrations [8,13,22,102,103]. Among these components, typical *Populus* propolis usually contains mainly benzoic acid and its esters (mainly benzyl benzoate) and oxygenated sesquiterpenoids (*α* and *β* and *γ*-eudesmols) [13,22,88].

Despite the fact of the known antibacterial activity of many singular components as well as the whole propolis EOs, their impact on antibacterial activity is unknown due to their evaporation during the preparation of extracts used in most antibacterial research. However, some semi-volatile antibacterial components of propolis EOs may be still present in dry extracts, especially benzoic acid and its esters [88]. Moreover, previous propolis liquid extracts, which are rich in volatile components, are often used in medicine. For these reasons, the impact of EOs on *Populus* propolis antibacterial activity should be further investigated.

## 5. Mechanism of Antibacterial Action of Propolis

It was found that poplar propolis exhibits multifactorial mechanisms of action. Propolis works on bacterial cell barriers on different ways:

- disruption of function and disintegration of bacterial cell barriers,
- inhibition of DNA and protein synthesis,
- inhibition of energy synthesis,
- induction of oxidative stress,
- disruption of quorum quenching as well as biofilm formation,
- induction of human defense system and other indirect effects.

All these processes relate to each other, and it is difficult to discuss them in isolation. For this reason, they are described in three paragraphs: the direct effect of propolis on bacterial cells, the impact of poplar propolis on bacterial biofilms and colonies and the indirect antibacterial effects of poplar propolis.

### 5.1. Direct Effect of Poplar Propolis on Bacterial Cells

The best documented effect of propolis is impact on cell barriers. It is known that propolis extracts and its components disrupt membrane function and increase membrane permeability [52]. This effect depends on the bacterial species and propolis concentration [52]. Disruption of the cell membrane leads to ion loss from bacterial cells, uncouples the energy transducing cytoplasmic membrane and inhibits bacterial motility. In higher concentrations, propolis extracts may lead to lysis of bacterial cells. The impairment of bacterial membrane integrity and increase in permeability impair essential functions of bacterial cells, such as ATP production and nutrient transport. The reduction of ATP synthesis is achieved via the reduction of membrane potential through the induction of changes in the permeability of the cell membrane, which in turn causes disruption of the proton pump and consequently reduction of ATP (adenosine-5-triphosphate). These processes may lead to reduced mobility or even complete immobilization of ciliated bacteria. This in turn ultimately compromises bacterial viability and consequently the ability of bacterial cells to form biofilms. It is worth noting that propolis generally impacts Gram-positive bacteria more strongly than Gram-negative ones. It may be due to the presence of hydrolytic enzymes in the outer membrane of Gram-negative bacteria, which can inactivate or degrade flavonoids, phenolic acids, or other bioactive compounds from propolis, in effect reducing its inhibitory effect on bacterial growth [104,105]. These effects have been demonstrated for propolis extracts [52] and components such as quercetin [52], galangin [92,106] and CAPE (caffeic acid phenethyl esters) [52]. The most common propolis action on cell barriers relates to the presence of flavonoid aglycones [52]. Research [107] suggests that flavonoids may interact with the hydrophilic region of phospholipids of the membrane in lower concentrations and with the hydrophobic core in higher ones. As a result, ion canals in bacterial cell membranes may be created, which leads to the uncontrolled loss of ions, disruption of bacterial metabolism, and lysis of cells. Moreover, some polyphenols of propolis may work as ionophores [52], especially flavonoids, which increase disruption of ion balance in cells. Typical flavonoid aglycones for worldwide propolis (mainly poplar) are chrysin, tectochrysin, pinocembrin, pinobanksin, pinobanksin 3-acetate, pinostrobin, apigenin, naringenin and galangin [29,50,108,109,110,111]. However, there are also known specific flavonoid aglycones with prenyl or polyprenyl chains. Due to the presence of lipophilic prenyl chains, this form of flavonoid may work more strongly on bacterial cell barriers [92,112]. These components are present in propolis of the “Pacific” type, which occurs in the Pacific regions of Asia, such as Taiwan [113], the Solomon Islands (solophenols) [114], Egyptian [115] and Indonesian propolis [40]. Apart from polyphenols, propolis also contains other compounds which also disrupt the function of cell barriers. Among them, probably the most important are terpenoids. These substances are part of the essential oils and non-volatile fraction of propolis. Due to their lipophilic nature, they may dock into a phospholipid bilayer and disrupt its function [116]. Such hydrophobic interactions have been reported to cause alterations of membrane proteins, potassium ion leakage, and in effect lead to cellular collapse [104]. Moreover, some of them also exhibit additional effects, such as the inhibition of the efflux pump in the case of totarol [117], which increase the antibacterial effects of other components.

Another mechanism of propolis antibacterial action is the inhibition of protein synthesis. This activity is usually connected with the presence of flavonoids and hydroxycinnamic monoesters. For example, flavonoids inhibit DNA gyrase [118,119,120,121,122] and modify the action of topoisomerase IV [118,123,124], while CAPE works as an inhibitor of peptide deformylase of *H. pylori* [125]. This enzyme is necessary for the initial part of translation (removing the formyl group from the nitrogen bond of N-formylmetionine, the first amino acid in the translation process). Other studies showed that propolis components inhibit bacterial DNA-dependent RNA polymerases and restrict the endonuclease of *Escherichia coli* and *Streptomyces aureofaciens* [126]. Inhibition of protein synthesis by propolis leads to malfunctions in bacterial metabolisms and cell regenerative mechanisms. As a result, the repair of direct damage is impossible and bacterial cell lysis results.

Apart from DNA, RNA and protein synthesis, propolis also exhibits impacts on bacterial energy synthesis. This process is disrupted by direct damage to mitochondrial membranes and the inhibition of enzymes connected with ATP synthesis and the maintenance of oxidation-reduction balance in bacterial cells. For example, flavonoid aglycones affect the activity of various enzymes, leading to metabolic disorders. For example, some chalcones can inhibit cytochrome C reductase, while there was no observed inhibition of cytochrome C oxidase or NADH-CoQ reductase [127]. As a result, ATP synthesis as well as the oxidation-reduction balance in the cell is disrupted. The inability to neutralize free radicals in bacteria causes oxidative and other damage.

### 5.2. Impact of Poplar Propolis on Bacterial Biofilms and Colonies

Bacterial biofilms are organized microbial communities attached to biotic or abiotic surfaces and embedded in a self-produced extracellular polymeric substance (EPS) matrix composed mainly of polysaccharides, proteins, and extracellular DNA (eDNA), and they play a crucial role in the pathogenesis and persistence of chronic infections [128]. The transition from planktonic growth to a sessile phenotype markedly increases bacterial tolerance to antibacterial agents and enhances protection against a host’s immune defense mechanisms. Therefore, the antimicrobial activity of propolis extracts against planktonic cells cannot be directly extrapolated to their efficacy against biofilm-associated infections. Numerous studies indicate that propolis extracts can affect multiple stages and processes involved in biofilm development and persistence, including initial attachment and surface colonization, cell–cell aggregation, extracellular matrix production, biofilm maturation, and the expression of adhesion-related virulence factors. At sufficiently high concentrations, some extracts may also reduce the viability of cells embedded in mature biofilms. This broad pattern is consistent with the chemical complexity of propolis and suggests that its antibiofilm activity may involve effects on bacterial membranes, cell surface physicochemical properties, motility, quorum sensing, and matrix production, rather than action on a single molecular target.

The antibiofilm activity of *Populus*-type propolis has been investigated most extensively in *Staphylococci*. An ethanolic extract of Polish propolis inhibited biofilm formation by *Staphylococcus epidermidis* strains at concentrations of 0.39–1.56 mg/mL, with the extent of inhibition depending on both extract concentration and exposure time [129]. A study of another Polish ethanolic extract of propolis (EEP) highlighted the distinction between inhibiting planktonic growth and reducing cell viability within mature biofilms. The extract exhibited an MIC of 128 µg/mL against planktonic *Staphylococci* and an MBEC_50_ of 128 µg/mL, whereas concentrations two to four times higher were required to achieve an estimated 90% reduction in viable cells within mature *S. epidermidis* biofilms. Even at these concentrations, the EPS matrix remained partially intact, and a residual population of viable bacteria persisted [130]. These findings suggest that a substantial reduction in bacterial viability does not necessarily entail complete disruption of the biofilm scaffold. They therefore provide a rationale for investigating combinations of propolis extracts with agents that target matrix components, such as polysaccharide intercellular adhesin (PIA), proteins, or eDNA [130].

A study evaluating a Portuguese ethanolic propolis extract against 45 biofilm-producing staphylococcal isolates demonstrated inhibition of biofilm formation and a reduction in the biomass of established biofilms. The mean inhibition of biofilm formation was 71%, while the mean reduction in preformed biofilm biomass was 88.5% [131]. The extract had a total phenolic content of 67.6 mg gallic acid equivalents (GAE)/g of dry extract and a total flavonoid content of 54.8 mg quercetin equivalents (QE)/g of dry extract. Moreover, UPLC–QTOF–MS/MS analysis indicated the presence of pinobanksin, chrysin, acacetin, apigenin, pinocembrin, and kaempferol dimethyl ether [131]. The relationship between chemical composition and antibiofilm activity varied depending on the endpoint assessed. Previous studies reported associations between total phenolic, flavonoid, and condensed tannin contents and inhibition of biofilm formation. However, condensed tannin content was positively associated with a reduction in preformed biofilm biomass, whereas total flavonoid and total phenolic contents showed inverse associations with this endpoint [131]. At the level of individual compounds, apigenin, pinocembrin, and kaempferol dimethyl ether were associated with greater reductions in preformed biofilm biomass, but not with inhibition of biofilm formation, whereas pinobanksin, chrysin, and acacetin exhibited a different pattern of associations [131]. Thus, total polyphenol content alone may be insufficient to predict antibiofilm activity; the relative proportions of individual compounds and their potential interactions may also contribute to the observed effects.

Initial adhesion is a key stage in staphylococcal biofilm formation that can be affected by propolis. A Spanish ethanolic propolis extract reduced adhesion and biofilm formation by *S. epidermidis* strains by up to 90.5% at subinhibitory concentrations [128]. This effect was accompanied by changes in the physicochemical properties of the bacterial cell surface. Increasing extract concentrations generally shifted the cellular zeta potential toward more negative values and tended to increase cell surface hydrophobicity. At the highest concentrations tested, differences in these properties between strains with different biofilm forming capacities were reduced [128]. The authors proposed that amphiphilic constituents of propolis progressively accumulate at the bacterial cell surface through intermolecular interactions, with their aromatic moieties oriented toward the surrounding medium. Such changes in cell surface properties may alter the electrostatic and hydrophobic interactions involved in attachment to biomaterials. This proposed mechanism may be particularly relevant to *S. epidermidis*, in which polysaccharide intercellular adhesin (PIA) and surface proteins contribute to cell–cell aggregation and adhesion to surfaces [128].

Recent studies provide evidence that propolis modulates the expression of genes involved in staphylococcal adhesion and extracellular matrix production. In methicillin-resistant *Staphylococcus aureus* (MRSA), an ethanolic extract of propolis reduced biofilm biomass and metabolic activity, inhibited cell adhesion and aggregation, and decreased extracellular polysaccharide and protein production [79]. These effects were accompanied by reduced expression of sarA, genes of the icaADBC operon, and the adhesin-encoding genes fnbA, fnbB, clfA, and clfB [79]. The icaADBC operon encodes proteins involved in the synthesis and processing of polysaccharide intercellular adhesin (PIA), whereas fnbA and fnbB encode fibronectin-binding proteins and clfA and clfB encode clumping factors involved in attachment and aggregation. SarA is a global regulator of staphylococcal virulence that also promotes biofilm formation. Thus, reduced expression of these genes may help explain the observed decreases in extracellular matrix components, adhesion, and biofilm formation.

Caffeic acid phenethyl ester (CAPE), a characteristic phenolic acid ester found in poplar-type propolis, may contribute to its antibiofilm activity. CAPE inhibited MRSA biofilm formation, reduced bacterial adhesion, and decreased the levels of both water-soluble and alkali-soluble extracellular polysaccharides [132]. RT-qPCR analysis revealed significant reductions in the expression of genes within the icaADBC operon, as well as sarA, fnbA, fnbB, clfA, and clfB, consistent with the expression changes reported for the whole propolis extract [132]. Because CAPE is abundant in many poplar-type propolis samples and has been reported to reproduce several key antibiofilm effects observed with whole propolis extracts, it may be an important contributor to their activity. Nevertheless, CAPE did not completely prevent biofilm formation under the conditions tested. Other constituents may also contribute to the activity of the whole extract, although their individual effects and potential interactions with CAPE require further investigation.

Evidence from Gram-negative bacteria further indicates that multiple propolis constituents possess antibiofilm activity. In *Klebsiella pneumoniae*, propolis and four propolis-derived compounds—naringenin, taxifolin, syringic acid, and gallic acid—exhibited concentration-dependent antibiofilm effects [133]. The whole propolis preparation showed the highest overall efficacy, achieving 79.1% inhibition of biofilm formation at twice its MIC, while the individual compounds reduced bacterial adhesion by approximately 50–81% and disrupted extracellular matrix architecture [133]. Taxifolin showed the strongest predicted binding to OmpA among the compounds tested and increased the proportion of non-viable cells within the biofilm. Propolis, naringenin, taxifolin, and gallic acid also reduced the expression of fimH, ompA, mrkA, and mrkD, genes associated with adhesion, outer membrane functions, and fimbrial structure or activity [133]. The presented findings suggest that individual polyphenols may act on specific biofilm-associated proteins or regulatory pathways, while the greater overall activity observed for the whole propolis extract may reflect contributions from multiple constituents acting through different mechanisms.

In *Pseudomonas aeruginosa*, the antibiofilm activity of propolis may involve effects on extracellular matrix production and bacterial motility. Italian propolis extracts and *Populus* bud resin extracts reduced biofilm formation at sub-MIC concentrations and decreased the number of viable biofilm-associated cells [134]. Both preparations partially inhibited swimming motility, potentially limiting the ability of bacteria to overcome repulsive forces during reversible attachment to a surface. The poplar bud resin extract also increased swarming motility, which the authors interpreted as potentially interfering with progression toward a mature biofilm, whereas twitching motility remained unaffected [134].

In another study of Albanian propolis, the ethanolic extract showed greater antibiofilm activity than preparations obtained using propylene glycol or polyethylene glycol 400 (PEG 400). It also reduced biofilm formation, eDNA release, and phenazine production [135]. Because eDNA is an important structural component of the *P. aeruginosa* extracellular matrix and phenazines contribute to redox homeostasis, signaling, and biofilm physiology, the observed reductions in eDNA release and phenazine production suggest that propolis may affect biofilm-associated processes in addition to bacterial viability. The phytochemical profile of the Albanian extract also identifies potential contributors to its antibiofilm activity. Chrysin, galangin, pinocembrin, and pinobanksin derivatives were among the major flavonoids, while caffeic acid derivatives, including CAPE, were also abundant [135]. Following incubation of the extract with *P. aeruginosa*, chromatographic analysis showed consistent decreases in the peak areas corresponding to quercetin and CAPE, with smaller decreases observed for pinobanksin-3-acetate and pinobanksin-3-butyrate [135]. The authors interpreted the decreases in quercetin and CAPE levels as suggesting possible interactions with bacterial cells. Quercetin has been reported to disrupt bacterial membranes and inhibit extracellular protein function, whereas CAPE and other caffeic acid derivatives have been reported to interfere with bacterial replication [135]. Moreover, pinocembrin and apigenin have previously been shown to reduce biofilm thickness and alter biofilm architecture in *Streptococcus mutans* [135]. These findings suggest that several polyphenols characteristic of poplar-type propolis contribute to its antibiofilm activity, with their relative contributions potentially varying depending on the bacterial species and stage of biofilm development.

Oral biofilms provide another example of the effects of a chemically distinct propolis fraction on multiple virulence determinants. Analysis of an essential oil obtained from Chinese poplar-type propolis (PEO) identified 79 constituents, including β-himachalene (13.94%), α-curcumene (11.28%), α-bergamotene (4.50%), sesquicineole (4.35%), and β-bisabolene (3.00%) among the major components [136]. PEO reduced the viability of *S. mutans* cells within biofilms, disrupted biofilm architecture, and increased the release of lactate dehydrogenase (LDH) and Ca^2+^, consistent with compromised cell membrane integrity [136]. PEO also reduced cell adhesion, decreased the levels of both water-soluble and water-insoluble EPS, and inhibited glucosyltransferase (GTF) activity [136]. Because GTFs synthesize extracellular glucans that contribute to bacterial attachment and the accumulation of cariogenic dental plaque, the observed inhibition of GTF activity links the effects of the volatile propolis fraction to a specific virulence-associated process. These findings indicate that antibiofilm activity is not restricted to the non-volatile polyphenolic fraction: a terpenoid-rich propolis essential oil can also compromise membrane integrity, reduce adhesion, and decrease extracellular polysaccharide levels.

Interference with quorum sensing may also contribute to the antibiofilm activity of propolis. Propolis has been reported to inhibit signaling mediated by N-acyl homoserine lactones (AHLs) [137]. Algerian ethanolic propolis extracts produced concentration-dependent inhibition of violacein production in *Chromobacterium violaceum*, a commonly used reporter organism for quorum-sensing assays [138]. The more active extract, obtained from Guelma, had higher total phenolic and flavonoid contents. Cynarin was the major identified compound, while caffeic acid, naringenin, and several other flavonoids were also detected [138]. The same extract showed the strongest activity against a multidrug-resistant (MDR) *S. aureus* strain and reduced preformed biofilm biomass by approximately 60% at the highest reported level [138].

However, the study did not establish that cynarin itself was responsible for the observed antibiofilm or quorum-sensing inhibitory effects. Its abundance therefore represents a compositional observation rather than direct evidence of a causal role. Potential synergistic or antagonistic interactions among extract constituents further complicate the attribution of activity to individual compounds whose contributions cannot be inferred from their concentrations alone.

Formulation may also influence the antibiofilm activity of propolis. The Malaysian propolis investigated contained quercetin, luteolin, kaempferol, apigenin, and pinocembrin, with kaempferol and pinocembrin showing the highest concentrations among the quantified flavonoids [139]. Chitosan–propolis nanoparticles reduced bacterial counts in *E. faecalis* biofilms by approximately 90% at 200 µg/mL and disrupted preformed biofilms by approximately 40% and 75% at 200 and 300 µg/mL, respectively [139].

Taken together, the available evidence suggests that propolis exerts antibiofilm activity through multiple mechanisms. The reported effects include disruption of bacterial membrane integrity, changes in cell surface charge and hydrophobicity, reduced swimming motility, inhibition of adhesion, and GTF activity, decreased extracellular polysaccharide levels, reduced eDNA release and phenazine production, interference with quorum sensing, and downregulation of biofilm-associated genes. Evidence for these effects varies across preparations and experimental models. Among individual constituents, CAPE has molecular evidence supporting its antibiofilm activity in MRSA, while quercetin, pinocembrin, apigenin, taxifolin, naringenin, and gallic acid have shown antibiofilm effects in selected bacterial models [138]. However, neither total phenolic content nor the abundance of an individual compound consistently predicts whole-extract activity [130]. This variability may reflect differences in chemical composition, potential interactions among constituents, and variation in bacterial species, biofilm maturity, and experimental methods. Mature biofilms can remain difficult to eradicate, and substantial reductions in the viability of biofilm-associated cells do not necessarily entail complete disruption of the extracellular matrix bacteria [130,132,133,135]. Consequently, standardized propolis preparations enriched in well-characterized active compounds, or combinations with EPS-degrading enzymes, nanoparticles, or conventional antibiotics, appear to be the most rational directions for the development of propolis-based antibiofilm strategies.

### 5.3. Indirect and Additional Effects

The last antibacterial effect of poplar propolis is the impact of propolis on the human immune system. Research has demonstrated the immunostimulant effects [140] of propolis. These activities include: macrophage and neutrophil activation and stimulation of antibody production [140]. All these effects activate the human organism’s self-defense against microorganisms and help to prevent and treat infections. Researchers usually connect them with CAPE and other esters of hydroxycinnamic acids [140]. However, these components are also important in reducing the inflammatory processes induced by bacteria. It was found in tests with rats that CAPE reduces kidney damage resulting from oxidative stress induced by *Escherichia coli.* This may have clinical relevance in the treatment of bacterial nephropathy [141]. Moreover, another research study tested the activation of the NF-κB pathway [142]. As a result, the unnecessary tissue damage caused by the human’s own defense system is limited. However, further research is required to explain this process and evaluate its reproducibility.

## 6. Propolis and Antibiotics

Recent peer-reviewed studies show that *Populus* propolis can enhance the antimicrobial efficacy of conventional antibiotics through several biochemical and microbiological mechanisms. A similar phenomenon was also reported for other propolis types, such as Brazilian green propolis [143,144,145]. This is important, as multidrug-resistant Gram-positive pathogens including methicillin-resistant *Staphylococcus aureus* (MRSA) and vancomycin-resistant Enterococcus (VRE) strains are now considered the common cause of severe and life-threatening infections. While currently used antibiotics are individual substances usually acting on one metabolic pathway, propolis is a multicomponent mixture and exhibits multidirectional action. It is likely that this is the reason why *S*. *aureus* cannot create any mutants resistant to propolis extract [146]. However, MRSA usually exhibits lower susceptibility to propolis extract than methicillin-susceptible *S. aureus* (MRSA) [77,146]. In contrast, Gram-negative bacteria display substantially lower susceptibility to propolis as a result of both a different composition of the bacterial membrane and the development of other efflux mechanisms [147,148]. Moreover, it should be emphasized that a synergistic effect against various bacteria was observed for subinhibitory doses of propolis extracts [52,80,149]. This may further prove that the synergism between propolis and antimicrobial drugs is an effect of inhibition or deregulation of bacterial resistance mechanisms rather than antibacterial activity.

Among the many possible mechanisms of interaction of bee glue and antibiotics, three main categories can be distinguished.

Synergistic antimicrobial activity is the first of them. As propolis contains polyphenols, mainly flavonoids (chrysin, galangin, quercetin) and phenolic acids (caffeic, ferulic, *p*-coumaric) it is capable of disrupting bacterial membranes, causing enzyme inhibition, and interfering with nucleic acid synthesis. These effects can synergize with antibiotics and therefore increase bacterial membrane permeability and inhibit resistance enzymes, thereby improving drug uptake and reducing resistance [150]. Modulation of bacterial resistance mechanisms is the second mechanism of interaction. The flavonoid and phenolic components of propolis can suppress efflux pump activity and reduce β-lactamase activity. As those mechanisms are often responsible for antibiotic resistance, said resistance can be lowered by using propolis as an adjuvant that enhances antibiotic action at lower doses. Target overlap with antibiotics is the third possible mechanism. Propolis compounds often affect bacterial cell wall synthesis and DNA replication, similar to antibiotics such as β-lactams and quinolones. Hence, co-administration tends to yield synergy, particularly with antibiotics targeting cell wall (e.g., vancomycin, oxacillin) or protein synthesis (e.g., macrolides) rather than nucleic acid synthesis [71,143].

It is worth noting that bee glue can interact with many antibiotics in different ways. The type of effect (synergism, antagonism, no interaction effect) depends on the species of bacteria, antibiotics used, and the propolis composition.

Agonism was also observed with the use of propolis against *Staphylococcus* spp. (including *Staphylococcus aureus*) in combination with numerous antibiotic groups: β-lactams, tetracyclines, aminoglycosides, lincosamide, chloramphenicol, macrolids, vancomycin, polymyxin [77,80,151]. The moderate synergy was observed in most cases. However, the synergistic effect of these antibiotics is not a rule, and some investigators reported a lack of effect. This problem includes some antibiotics, chiefly erythromycin [80], chloramphenicol, ciprofloxacin [77].

The combination of ampicillin with propolis against *S. aureus* gave diverse results, from strong synergism [80] to a lack of effect [151]. Data showed that for β-lactams, aminoglycosides, and nalidixic acid, a synergistic effect was also observed for *Bacillus subtilis* [52].

It was reported that checkerboard dilution of two-drug combinations between ethanol or aqueous extracts of propolis with antibiotics (vancomycin, oxacillin, and levofloxacin) demonstrated synergistic interaction against microorganisms, including *Streptococcus pyogenes*. A combination of ethanol propolis extract and levofloxacin revealed a synergistic interaction against fastidious human respiratory bacterial pathogens including *Streptococcus pneumoniae* and *Haemophilus influenzae* [71]. Those results were further confirmed by a time-kill–kinetic assay of two-drug combinations between ethanol propolis extracts and water propolis extracts with antibiotics. The two-drug combination of sub-MIC ethanol propolis extract with sub-MIC of vancomycin against *Streptococcus pyogenes*, VRE and MRSA displayed synergistic interactions with bacteriostatic effects and reductions in colony count after overnight incubation compared to vancomycin as the reference drug. Analogous results were reported for oxacillin and levofloxacin against the fastidious bacteria *H. influenzae* and *S*. *pneumoniae* [71].

It is noteworthy that a synergistic action of propolis with certain antibiotics against Gram-negative bacteria was also observed. *E. coli* was found to be sensitive to tetracycline and nalidixic acid in combination with propolis [52]. However, the number of antimicrobial drugs acting synergistically with propolis extract against Gram-negative bacteria was significantly smaller than the number that act so against *Staphylococcus aureus*. Additionally, the synergistic effect of antibiotic–propolis combinations was weaker against Gram-negative bacteria. However, ampicillin and doxycycline combined with propolis showed agonistic antibacterial effects against *Klebsiella pneumoniae* [52].

On the other hand, synergism between β-lactam antibiotics, including amoxycillin, ampicillin, cephalexin and propolis (from Bulgaria and Brazil) was also observed against *Salmonella enterica* serovar Typhi [144]. However, further investigation [145] did not confirm the synergistic effect for co-trimoxazole, ciprofloxacin, or norfloxacin used together with Brazilian and Bulgarian bee glue against *Salmonella typhi*. The subsequent studies conducted by these researchers revealed that combinations of Bulgarian propolis with chloramphenicol, tetracycline, and neomycin acted synergistically and showed bactericidal activity against *Salmonella typhi*, whereas Brazilian propolis exhibited only bacteriostatic activity [152].

A synergistic action was demonstrated for the combination of propolis with clarithromycin against *Helicobacter pylori* [153]. However, no synergistic effect was observed for the β-lactam antibiotics, macrolides, and fluoroquinolones combined with propolis against *Streptococcus pneumoniae*, *Haemophilus influenzae*, *Haemophilus parainfluenzae*, *Moraxella catarrhalis* and *Streptococcus pyogenes* strains [154].

In the case of *Mycobacterium tuberculosis*, both synergism and antagonism were observed during the use of different antibiotics together with propolis. Scheller et al. [53] demonstrated that propolis increased the activity of rifampicin, isoniazid, and streptomycin but inhibited ethambutol. The authors suggested that it could be the result of formation of inactive derivatives as a result of a reaction of propolis extract with ethambutol. From a clinical point of view, there was a negative effect or lack of correlation between the virulence of the *Mycobacterium* and its sensitivity to the bee glue [53].

Investigations on an animal model revealed that a combination of ciprofloxacin with propolis acts synergistically in the treatment of eye infections in rabbits caused by *Pseudomonas aeruginosa* [155].

In addition, it was found that propolis can haver a good healing effect in therapy when combined with ciprofloxacin [155]. A synergistic effect was observed in combined therapy using propolis with mupirocin in treatment of rabbit nasal infections caused by *Staphylococcus aureus* MRSA [156] and with cefazolin in a rabbit arthritis model [157]. The combination of propolis with antibiotics showed synergism in the situation of an increase of bacterial resistance to antibiotics. It was observed that the use of extracts of propolis before and during antibiotic therapy can relieve the effects of the oxidizing stress and immunosuppression caused by oxytetracycline [158].

It has been also found that that propolis can enhance the antibacterial performance of nanostructured materials. A recent study described ZnO–propolis nanocomposites that had shown a stronger inhibition against *E. coli* and *Pseudomonas aeruginosa* than either ZnO nanoparticles or propolis alone, suggesting synergistic physical–chemical antibacterial effects. It has been found that both ZnO nanoparticles and ZnO nanocomposites disrupt bacterial cell membranes. It provides a good counter to antimicrobial resistance, which is highly important regarding the growth of antibiotic resistance, especially in light of antibiotic overuse and the resulting increased growth of resistance, as it provides a solution to multidrug-resistant bacteria [143].

However, there are certain limitations to the use of propolis as an adjuvant for antibiotics. Among the key limitations noted in existing studies, three are observed regularly. The most important impediment to application of propolis is the variability of propolis composition based on geographic origin and extraction method. It severely complicates attaining reproducibility of the antibiotic synergy results found in experiments. There is also a lack of standardized dosages and limited clinical data, as most of the studies remain in vitro, with only a few going to animal models or clinical trials. Finally, there is the possibility of potential allergic reactions and the unclear pharmacokinetics of propolis extracts. While those problems do not subtract from the value of the research into propolis’s interaction with antibiotics regarding antimicrobial effects, further studies and safety evaluations are required before clinical integration [150].

## 7. Practical Applications of Propolis

### 7.1. Nanotechnology in Applications of Propolis

Nanotechnology represents an emerging strategy for overcoming some of the intrinsic limitations associated with conventional propolis formulations. Despite its broad antimicrobial potential, the practical application of propolis may be limited by poor aqueous solubility, limited bioavailability and absorption, and non-specific release of its active constituents. Nanoencapsulation and other nanoscale delivery approaches can improve dispersion, protect phenolic and flavonoid constituents, enhance interactions with biological surfaces, and potentially provide more efficient delivery of antimicrobial compounds [159,160]. Nano-propolis has therefore emerged as a particularly promising natural antimicrobial system, although its biological performance depends strongly on the physicochemical characteristics of the nanoparticles and the formulation strategy used. Seven et al. emphasized that nanosizing may improve the solubility and absorption of propolis and that nano-propolis frequently exhibits stronger antibacterial and antifungal activity than conventional propolis [159].

Particle size is one of the key parameters influencing the antibacterial properties of propolis-based nanosystems. Reduction to the nanoscale substantially increases the surface-area-to-volume ratio and consequently the area available for interaction with microorganisms. In general, small nanoparticles can penetrate bacterial cell walls and biofilm matrices more efficiently, thereby improving the access of antimicrobial agents to microbial cells [161]. The available literature on nano-propolis, however, demonstrates substantial variability in particle size. Formulations smaller than approximately 175 nm have shown enhanced activity against *Bacillus subtilis*, *Staphylococcus aureus*, *Escherichia coli*, and *Salmonella* spp., whereas other preparations ranged from approximately 100–322 nm, 175–873 nm, or around 300 nm [159]. In another studies, nano-propolis exhibited approximately two-fold higher antibacterial activity than the corresponding conventional propolis preparation [159]. Nevertheless, the currently available studies do not establish a direct quantitative relationship between particle size and antimicrobial potency. Differences in propolis origin, chemical composition, carrier material, preparation procedure, and microbiological methodology considerably limit direct comparisons between formulations.

The importance of surface morphology and particle architecture is particularly evident in recently developed propolis–carboxymethyl cellulose (CMC) Janus nanoparticles (anisotropic or biphasic nanoparticles with chemically distinct surface domains). Razavi et al. obtained particles with an average diameter of approximately 13.4 nm, with more than 99% of particles below approximately 37.7 nm [162]. The particles displayed a cubic morphology with rough surfaces, resulting in a relatively large specific surface area [162]. Their asymmetric Janus architecture generates chemically distinct domains, allowing simultaneous interactions with hydrophobic membrane components and hydrophilic extracellular environments. Such an amphiphilic architecture may facilitate interaction with lipid-rich bacterial membranes while maintaining compatibility with aqueous surroundings and surface proteins, thereby increasing membrane permeability and cellular damage [162]. Propolis–CMC Janus nanoparticles exhibited antibacterial activity against both Gram-positive *Listeria monocytogenes* and Gram-negative *E. coli*, with MIC values of 1.5 and 2.0 mg/mL, respectively. Their activity was concentration-dependent, and inhibition zones greater than 17 mm were observed at higher concentrations [162]. These findings suggest that antibacterial effectiveness is determined not only by particle diameter but also by surface topology and the spatial arrangement of hydrophilic and hydrophobic domains.

Another important determinant is propolis loading and encapsulation efficiency. Nanoencapsulation can improve the physicochemical stability and dispersibility of propolis constituents and may allow their sustained or controlled release. Several carrier systems have been explored, including maltodextrin, casein micelles, chitosan, alginate, gelatin, poly(ε-caprolactone), β-cyclodextrin and protein–polysaccharide complexes [159]. For example, encapsulation of Indonesian propolis in casein micelles produced nano- and microparticles with reported encapsulation efficiencies of approximately 94% for flavonoids and 67% for polyphenols [159]. The amount of biologically active material incorporated into the carrier, together with its subsequent release kinetics, should therefore be considered fundamental parameters influencing the effective antimicrobial concentration. However, systematic studies directly correlating loading efficiency, release kinetics, particle size and MIC/MBC values remain scarce.

The antibacterial properties of propolis-containing nanoparticles are probably mediated by multiple complementary mechanisms. Nanoformulation increases contact between bioactive constituents and bacterial surfaces, whereas propolis-derived phenolics and flavonoids can interfere with bacterial membranes, membrane-associated enzymes, energy metabolism, nucleic acids, and biofilm formation [159,162]. In the propolis–CMC Janus system, the combination of nanoscale dimensions, large surface area, and asymmetric surface architecture was proposed to facilitate bacterial membrane penetration and increase membrane permeability. Oxidative stress and reactive oxygen species generation may provide an additional mechanism contributing to cellular injury [162]. Thus, nanostructuring appears to potentiate rather than replace the intrinsic multitarget antimicrobial activity of propolis.

A conceptually different but highly promising approach involves the combination of propolis with intrinsically antimicrobial nanoparticles, particularly silver nanoparticles (AgNPs). Erdoğan et al. recently evaluated Turkish propolis in combination with AgNPs with an average diameter of 20 ± 4 nm [163]. Strong synergistic antibacterial effects were observed against carbapenem-resistant *Acinetobacter baumannii* and *E. coli*. Fractional inhibitory concentration index (FICi) values were 0.375 and 0.50, respectively, confirming synergistic interactions [163]. The MIC of propolis against *A. baumannii* decreased from 128 µg/mL when tested alone to 16 µg/mL in combination, whereas for *E. coli* it decreased from 256 to 64 µg/mL [163].

Importantly, this synergistic effect also extended to preformed bacterial biofilms. The propolis–AgNP combination inhibited *A. baumannii* biofilms by 81.3%, 72.6%, and 55.4% at 2 × MIC, MIC, and ½ × MIC, respectively, compared with 57.3%, 44.1%, and 38.5% for propolis alone. Corresponding inhibition of *E. coli* biofilms reached 76.4%, 65.7%, and 50.4%, compared with 54.7%, 42.2%, and 35.6% for propolis alone [163]. The superior antibiofilm activity of such combinations may arise from complementary mechanisms involving increased bacterial membrane interaction, disruption of the extracellular polymeric matrix, impairment of cell wall integrity, reactive oxygen species-mediated damage, and, in AgNP-containing systems, silver-ion-associated effects [163].

The potential importance of nanotechnology in oral and biofilm-related infections is also supported by the broader nanomaterials literature. Nanoparticles possess a large specific surface area, enhanced chemical reactivity, and the ability to penetrate microbial biofilms and otherwise poorly accessible biological structures [161,164]. Nanocarriers may additionally protect antimicrobial compounds against degradation and enable controlled release within the biofilm environment [161]. These properties make propolis-based nanomaterials particularly attractive for local antimicrobial applications, including oral, dental, wound-related, implant-associated, and other biofilm-related infections.

In *S. epidermidis*, a cationic chitosan–propolis nanoparticle formulation markedly disrupted biofilms and reduced the expression of genes within the icaADBC operon, as well as embp and several other genes involved in adhesion and biofilm development. The nanoparticles were more effective than the corresponding unformulated ethanolic or ethyl acetate propolis extracts [165]. These findings do not establish that kaempferol or pinocembrin individually account for the enhanced activity, as the chitosan carrier may also contribute to the observed effects. Rather, they support the potential of nanoparticle formulations to enhance the antibiofilm activity of propolis extracts, although improved delivery of propolis constituents to biofilm-associated cells would require direct confirmation. It is also possible that the inhibition of bacterial adhesion ability relates to the inhibition of virulent proteins such as lipase and coagulase in the case of *S. aureus*. The effect of inhibition of these enzymes is a reduction of adhesion ability of bacteria and biofilm formation [80].

Collectively, current findings identify propolis-based nanomaterials as a promising but still insufficiently standardized research area. Future studies should systematically investigate the relationships between particle size and size distribution, morphology, surface charge and chemistry, propolis loading, and encapsulation efficiency, release kinetics, colloidal stability, and antibacterial and antibiofilm performance. Particular attention should be given to standardized MIC, MBC, and MBEC assays, mature and multispecies biofilms, clinically relevant multidrug-resistant isolates, cytotoxicity and biocompatibility, and direct comparison with the corresponding non-nanoformulated propolis. Such studies would help determine whether improved antimicrobial performance results predominantly from enhanced delivery of propolis constituents, intrinsic activity of the nanocarrier, or genuine synergistic interactions between both components.

### 7.2. Limitation of Practical Application of Propolis

A fundamental limitation in research on propolis and its practical application is the instability of its composition. Consequently, unlike certain plant-based preparations, raw propolis does not hold the status of a medicinal product in pharmacopoeias. However, medicinal preparations based on standardized propolis extract are available on the market. The standardization process involves sourcing raw propolis from areas with similar flora and performing a quantitative and qualitative assessment of each extract batch. The extracts are then blended to yield a final product with a defined composition and predictable activity. Since antibacterial activity is not attributable to a single specific constituent, standardization requires assessing antibacterial activity [7,49] and measuring the content of specific compound groups (typically the total phenolic and flavonoid content) [7,49]. Individual constituents are also analyzed due to their association with biological activity or allergenic properties [7,49]. In the case of poplar propolis, the primary allergens are caffeic acid esters—specifically 1,1-dimethylallyl and phenethyl esters [7]. However, all these recommendations were prepared for poplar propolis types. It is unknown if aspen and aspen–poplar propolis should have the same standardization procedures or specific techniques should be prepared. As a result, standardization of *Populus* propolis remains a problem only partially solved.

While this enables the production of an effective and safe medicinal preparation from poplar propolis, its use is restricted to topical applications for various reasons. The vast majority of research into the antibacterial activity of poplar propolis consists of in vitro studies, and reliable data from in vivo studies remains limited. This applies particularly to clinical trials. Most available data date back to the period prior to the 1996 introduction of the first version of the ICH GCP (Good Clinical Practice) guidelines—specifically E6—established by the International Council for Harmonisation of Technical Requirements for Pharmaceuticals for Human Use. Furthermore, many of these studies were conducted in the former Soviet Union, and information regarding them is available only through citations in books. Various authors have reported on the efficacy of propolis preparations in treating skin infections caused by *S. aureus* and *S. pyogenes* and chronic bacterial diarrhea in children [166], as well as in the treatment of gastric and duodenal ulcers, chronic bronchitis and pneumonia, and the common cold, and as an adjunct therapy for pulmonary tuberculosis [167]. However, it should be noted that the reliability of these data is difficult to verify. On the other hand, clinical studies conducted between 1996 and 2026 have demonstrated the efficacy of various propolis preparations (poplar, non-poplar, and of unknown plant origin) as an adjunct to standard treatment for *H. pylori* infections [168], in the management of gingivitis and dental plaque [169,170,171,172,173,174], an alternate root canal filling material in the primary teeth [175], treatment of recurrent aphthous stomatitis [176,177], the improvement of sexual and urinary symptoms in participants with recurrent urinary tract infections [178], adjuvant treatment of uncomplicated cystitis in women [179,180,181], treatment of chronic vaginitis [182], remission of symptoms of uncomplicated upper respiratory tract infection [183], preventing acute otitis media in children [184], improvements in human diabetic foot ulcer healing [185], and nonhealing venous leg ulcers [186]. The main limitations of some these clinical studies included: unknown propolis composition (and plant origin), small patient group sizes, difficulty in determining the direct effect of propolis (when used in combination therapies), and short periods of clinical trials. Furthermore, in some cases, only commercial preparations were analyzed, without independent data being available. Similar to standardization, there are no direct clinical data about aspen and aspen–poplar propolis types.

Most clinical studies focused on dental and oral diseases and the treatment of various types of wounds. Therefore, the highest efficacy of propolis is to be expected in these areas. Activity in other fields may be of interest but requires further research, particularly regarding urinary tract infections. In studies concerning this area [179,180,181], propolis was used in combination with standard medications (particularly cranberry or ciprofloxacin), without comparison to preparations in which propolis served as the sole active pharmaceutical ingredient (API). The situation differs regarding clinical studies in dentistry, where there are reports free from this issue. If the cited clinical trials are limited only to a poplar propolis type of proven origin, its exhibited efficiency in treatment is limited to: gingivitis induced by dental plaque [172,174], uncomplicated upper respiratory tract infection (URTI) [183], and nonhealing venous leg ulcer [186]. Poplar origin may be suspected in research on the efficacy of propolis in preventing acute otitis media in children [184] and as an adjunct to standard treatment for *H. pylori* infections [168]. In any of the analyzed studies, aspen or aspen–poplar origin of propolis cannot be confirmed due to a lack of evaluation of aspen markers. It may have an efficacy similar to that of poplar propolis due to in vitro research. However, further clinical research is required for aspen and aspen–poplar propolis.

In addition to the inconclusive clinical trial results regarding the internal use of propolis, the pharmacokinetics of propolis itself and its constituents also present challenges. Due to the viscosity of propolis extracts, their complex composition, and traditional usage patterns, the oral route is preferred for internal administration. While intravenous or subcutaneous administration is possible for individual propolis constituents (or mixtures thereof), these routes remain largely theoretical due to the high cost of isolated components, their lipophilic nature, the risk of allergic reactions, and the lack of suitable pharmaceutical formulations. However, it is worth noting that certain constituents, such as CAPE, have demonstrated stability in human plasma, despite being unstable in rat plasma [187]. Such constituents (or their chemical analogues) could be considered candidates for future intravenously administered active pharmaceutical ingredients (APIs). In general, the oral bioavailability of individual propolis constituents derived from standard, unmodified extracts (e.g., ethanolic or water-ethanolic) is considered low [188]. Factors such as poor water solubility, slow dissolution rates, extensive first-pass metabolism, and degradation by gut microbiota result in only a small fraction of these substances reaching the bloodstream in an unchanged form. For instance, the bioavailability of chrysin has been estimated at less than 1% [189]. On the other hand, the bioavailability of certain commercial poplar propolis extracts was rated as high (57.16 ± 5.71% for a 400 mg extract dose and 43.82 ± 6.77% for a 1600 mg dose) when calculated in terms of cinnamic acid and flavonoid conjugates [190]. In the same study, flavonoids exhibited a bioavailability of approximately 15% in the form of parent compounds and/or phase II flavonoid metabolites [190]. For phenolic acids, this figure exceeded 100%, a result attributed to the likely conversion of flavonoids and phenolic acid esters into phenolic acids and their conjugates [190]. These studies demonstrated that, upon internal administration, the primary active components of propolis are phenolic acids rather than flavonoids or hydroxycinnamic acid monoesters. Phenolic acids exhibit weaker antibacterial activity than flavonoids and hydroxycinnamic acid monoesters [7]. Consequently, it can be hypothesized that propolis displays superior antibacterial properties when applied topically, as there is a lower likelihood of flavonoids and hydroxycinnamic acid monoesters being converted into potentially less active phenolic acid conjugates. However, confirming this hypothesis requires further research. In summary, given the aforementioned factors, the clinical use of propolis should be limited to standardized preparations used for mild external infections and/or as an adjunct to antibiotic therapy. However, the use of propolis to treat internal infections remains controversial and requires further in-depth research.

## 8. Conclusions

The prevalence of infections caused by antibiotic-resistant microorganisms has increased dramatically over the past few decades. The failure of conventional antimicrobial treatments is now the most important challenge for researchers and pharmaceutical industry. Therefore, there is an urgent need for new drugs and antimicrobial strategies. More and more studies have focused on the possibility of using natural substances which could potentiate the effect of conventional antimicrobial therapy, either when administered alone or in combination with classical antibiotics and chemotherapeutics.

Studies on propolis have been conducted for many years. Several beneficial health qualities of propolis have been widely described in the literature. However, the mechanism of action and connection of action with the *Populus propolis* composition is not yet well understood, especially for aspen and aspen–poplar propolis types. Many publications show that the chemical composition of propolis and its antibacterial activity depend on many factors such as local flora, time of collection, breed of bees, and many other factors. Because there is no possibility to control most of these factors, the composition of propolis is variable, which precludes the use of it as a medicine. On the other hand, the efficiency of standardized poplar propolis preparations has been proven in the treatment of mild diseases and external infectious diseases (affected on oral cavity and skin).

The antibacterial research on *Populus* propolis shows that it is a valuable antibacterial substance. Currently, most new antibacterial medicines are prepared as modifications of known and used medicines. Therefore, bacteria can very quickly develop resistance against those drugs. This does not apply to propolis, which is a complicated mixture of compounds with a very varied chemical structure. Therefore, propolis could be applied as an auxiliary substance when the bacteria exhibit significant resistance to antibiotics. As a result, the research on propolis and its antibacterial features could be helpful in development of new classes of antibacterial medicines. There is also a possibility to develop new mixtures of substances which support the action of currently used antibiotics. However, this way requires further clinical studies, especially for aspen and aspen–poplar propolis types, due to a lack of widely sufficient in vitro and vivo data.

## Figures and Tables

**Table 2:** Comparison of antibacterial activity of *Populus* species propolis against Gram-positive and Gram-negative bacterial strains based on minimal inhibitory concentration (MIC).

Bacteria Specie	Aspen Propolis	Aspen–Poplar Propolis	Poplar Propolis	Type of Investigated Extract
**Gram-positive strains**				
*Actinomyces naeslundii*	ND	ND	SS	96EEP [67]
*Bacillus cereus*	SS	SS, S, WS	SS, S, WS, R	70EEP [21,68,69], DEE [6], CE [70]
*B. subtillis*	WS	WS	SS, S, WS, R	70EEP [69,71,72], 80EEP [73,74], WEP [71]
*B. thuringiensis*	SS	ND	ND	DEE [6]
*Lactobacillus acidophilus*	ND	ND	S	96EEP [67]
*Lactobacillus* spp.	ND	ND	S, WS	70EEP [73,75]
*Listeria monocytogenes*	ND	ND	S, SW	80EEP [71,73]
*Micrococcus luteus*	SS	SS, S	SS	70EEP [21,68], 96EEP [59]
*Mycobacterium kanasi*	ND	ND	** R	** 95EEP [53,76]
*M. intracellulare*	ND	ND	** R	** 95EEP [53,76]
*M. tuberculosis*	ND	ND	** S, ** R	** 95EEP [53,76]
*M. xenopei*	ND	ND	** R	** 95EEP [53,76]
*Peptostreptococcus anaerobius*	ND	ND	SS	96EEP [53,76]
*P. micros*	ND	ND	SS	96EEP [53,76]
*Staphylococcus aureus*	SS, WS	SS, S, WS	SS, S, WS, R *	70EEP [21,54,68,69,71,72], 80EEP [73], DEE [6], CE [70], 96EEP [59], PG [54], DCh [18], WEP [71]
MLS_b_	WS	WS	S	70EEP [72]
MRSA	WS	WS	SS, S, WS	70EEP [64,71,72,74,77], 80EEP [78], 95EEP [79], WEP [71]
VRE	ND	ND	WS	70EEP [71], WEP [71]
*S. auricolaris*	ND	ND	WS	70EEP [80]
*S. capitis*	ND	ND	WS	70EEP [80]
*S. epidermidis*	SS, S	SS, S	SS, S, WS, R *	70EEP [21,68,69,71,80], 96EEP [59], DCh [18], WEP [71]
*S. haemolyticus*	ND	ND	WS	70EEP [78,80]
*S. hominis*	ND	ND	WS	70EEP [78,80]
*S. saprophyticus*	SS	ND	S, WS	DEE [6], 70EEP [71], WEP [71]
*S. schleiferi*	SS	ND	ND	DEE [6]
*S. warnerii*	ND	ND	WS	70EEP [80]
*Streptococcus agalactiae* (syn. *S. β-haemolyticus*)	ND	ND	SS, S, WS	70EEP [71,80,81], WEP [71]
*S. mutans*	ND	ND	SS, S, WS	70EEP [54,75], 96EEP [59], PG [54]
*S. oralis*	ND	ND	S, WS	70EEP [71], WEP [71]
*S. pneumoniae*	ND	ND	S, WS	70EEP [71,80], CE [70], WEP [71]
*S. pyogenes (GABHS)*	ND	ND	SS, S, WS, * R	70EEP [54,69,71], CE [70], PG [54], WEP [71]
*S. sorbinus*	ND	ND	SS	96EEP [59]
*S. thermophilus*	ND	ND	SS, S,	70EEP [71], WEP [71]
*S. viridans*	ND	ND	S, WS	70EEP [80]
*Enterococcus faecalis* (syn. *S. faecalis*)	S, R	S, WS, R	SS, S, WS, R	70EEP [21,68,69,72,80], 80EEP [73], 96EEP [59]
*Enterococcus casseliflavus*	ND	ND	WS, R	70EEP [71], WEP [71]
**Gram-negative strains**				
*Acinetobacter baumanii*	ND	ND	S, WS, R	70EEP [71,82], WEP [71]
*Aeromonas hydrophila*	ND	ND	WS, R	80EEP [73]
*Burkholderia cepacia*	ND	ND	WS, R	70EEP [71], WEP [71]
*Campylobacter jejuni*	ND	ND	S	80EEP [83]
*Escherichia coli*	WS, R	WS, R	* SS, * S, WS, R	70EEP [21,68,69,71,72,74], DEE [6], CE [70], *96EEP [59], DCh [18], WEP [71]
*Enterobacter cloacae*	ND	ND	WS, R	70EEP [71], DCh [18], WEP [71]
*Fusobacterium nucleatum*	ND	ND	S	96EEP [67]
*Haemphillus influenzae*	ND	ND	S, WS	70EEP [71], CE [70], WEP [71]
*Helicobacter pylori*	SS, S	SS, S	SS, S, * WS, * R	70EEP [21,54,55,68], 95EEP [58], EEP [84], PG [54]
*Klebsiella aerogenes* (syn. *Enterobacter aerogenes*)	ND	ND	SS	96EEP [59]
*K. oxytoca*	ND	ND	WS	70EEP [71], WEP [71]
*K. pneumoniae*	R	R	S, WS, R	70EEP [21,68,71,72,81,82], CE [70], DCh [18], WEP [71]
*Moraxella catarrhalis*	ND	ND	SS, S	CE [70]
*Porphyromonas* *gingivalis*	ND	ND	S	96EEP [67]
*Prevotella oralis*	ND	ND	S	96EEP [67]
*P.melaninogenica*	ND	ND	S	96EEP [67]
*Proteus mirrabilis*	R	WS, R	R	70EEP [21,68], CE [70]
*Pseudomonas aeruginosa*	S, WS, R	WS, R	* SS, S, WS, R	70EEP [21,68,69,71,72], DEE [6], CE [70], 96EEP [59], WEP [71]
*Salmonella choleraesuis*	ND	ND	WS, R	70EEP [71], WEP [71]
*S. enterica*	ND	ND	WS	70EEP [54], PG [54]
*S.* Typhimurium	WS	WS, R	* SS, S, WS, R	70EEP [21,68], * 96EEP [59]
*Shigiella flexneri*	ND	ND	S, WS, R	70EEP [71], 80EEP [73], WEP [71]
*Stenotrophomonas maltophilia*	ND	ND	S, WS	70EEP [82]
*Yersinia enterocolitica*	ND	ND	WS	70EEP [71], WEP [71],
*Veillonella parvula*	ND	ND	S	96EEP [67]
*Vibrio alginolyticus*	ND	ND	S, WS, R	MEP [85]
*V. cholerae*	ND	ND	SS, S	MEP [85]
*V. fluvialis*	ND	ND	S, WS, R	MEP [85]
*V. parahaemolyticus*	ND	ND	WS, R	MEP [85]
*V. vulnificus*	ND	ND	SS, S, WS	MEP [85]

**General abbreviations:** ND—there are no data or data was unclear (unspecified type of propolis); *—susceptibility level is non-typical for specified microorganism; **—MIC was not accurately determined and susceptibility/resistance criteria were based on authors’ claims; **Bacterial susceptibility abbreviations:** SS—strong susceptibility MIC < 100 [µg/mL]; S—middle susceptibility, 100 ≥ MIC < 500 [µg/mL]; WS—weak susceptibility, 500 ≥ MIC < 5 000 [µg/mL]; R—very weak susceptibility or resistance, MIC ≥ 5 000 [µg/mL]; **Extract abbreviations:** CE—unknown commercial extract; DCh—dichloromethane extract; DEE—diethyl ether extract; 70EEP—70% ethanol in water extract; 95EEP—95% ethanol in water extract; 96EEP—96% ethanol in water extract; EEP—absolute ethanol; MEP—absolute methanol extract; PG—100% propylene glycol extract.

## Data Availability

Data is available from corresponding author (piotr.okinczyc@umw.edu.pl).

## References

[1] Burdock G.A. (1998). Review of the biological properties and toxicity of bee propolis (propolis). Food Chem. Toxicol..

[2] Popova M.P., Bankova V.S., Bogdanov S., Tsvetkova I., Naydenski C., Marcazzan G.L., Sabatini A.G. (2007). Chemical characteristics of poplar type propolis of different geographic origin. Apidologie.

[3] Banskota A.H., Tezuka Y., Kadota S. (2001). Recent progress in pharmacological research of propolis. Phyther. Res..

[4] Przybyłek I., Karpiński T.M. (2019). Antibacterial properties of propolis. Molecules.

[5] Christov R., Bankova V., Tsvetkova I., Kujumgiev A., Delgado Tejera A. (1999). Antibacterial furofuran lignans from Canary Islands propolis. Fitoterapia.

[6] Isidorov V.A., Bakier S., Pirożnikow E., Zambrzycka M., Swiecicka I. (2016). Selective behaviour of honeybees in acquiring European propolis plant precursors. J. Chem. Ecol..

[7] Kaleta J. (2007). The Physicochemical Analysis of Propolis and the Possibility of Its Standardization [Analiza Fizykochemiczna Propolisu i Możliwości Jego Standaryzacji]. Dissertation Thesis.

[8] Bankova V., Popova M., Trusheva B. (2014). Propolis volatile compounds: Chemical diversity and biological activity: A review. Chem. Cent. J..

[9] Kujumgiev A., Tsvetkova I., Serkedjieva Y., Bankova V., Christov R., Popov S. (1999). Antibacterial, antifungal and antiviral activity of propolis of different geographic origin. J. Ethnopharmacol..

[10] Page M.J., McKenzie J.E., Bossuyt P.M., Boutron I., Hoffmann T.C., Mulrow C.D., Shamseer L., Tetzlaff J.M., Akl E.A., Brennan S.E. (2021). The PRISMA 2020 statement: An updated guideline for reporting systematic reviews. BMJ.

[11] Bankova V. (2005). Recent trends and important developments in propolis research. Evid.-Based Complement. Altern. Med..

[12] Okińczyc P., Widelski J., Nowak K., Radwan S., Włodarczyk M., Kuś P.M., Susniak K., Korona-Głowniak I. (2024). Phytochemical profiles and antimicrobial activity of selected *Populus* spp. bud extracts. Molecules.

[13] Okinczyc P., Szumny A., Szperlik J., Kulma A., Franiczek R., Zbikowska B., Krzyzanowska B., Sroka Z. (2018). Profile of polyphenolic and essential oil composition of polish propolis, black poplar and aspens buds. Molecules.

[14] Ristivojević P., Stević T., Starović M., Pavlović S., Özcan M.M., Berić T., Dimkić I. (2020). Phenolic composition and biological activities of geographically different type of propolis and black cottonwood resins against oral streptococci, vaginal microbiota and phytopathogenic *Fusarium* species. J. Appl. Microbiol..

[15] Ristivojević P., Trifković J., Gašić U., Andrić F., Nedić N., Tešić Ž., Milojković-Opsenica D. (2015). Ultrahigh-performance Liquid Chromatography and Mass Spectrometry (UHPLC-LTQ/Orbitrap/MS/MS) study of phenolic profile of Serbian poplar type propolis. Phytochem. Anal..

[16] Isidorov V.A., Szczepaniak L., Bakier S. (2014). Rapid gc/ms determination of botanical precursors of Eurasian propolis. Food Chem..

[17] Popova M., Trusheva B., Khismatullin R., Gavrilova N., Legotkina G., Lyapunov J., Bankova V. (2016). The triple botanical origin of Russian propolis from the Perm Region, its phenolic content and antimicrobial activity. Nat. Prod. Commun..

[18] Popova M., Giannopoulou E., Skalicka-Wózniak K., Graikou K., Widelski J., Bankova V., Kalofonos H., Sivolapenko G., Gaweł-Bȩben K., Antosiewicz B. (2017). Characterization and biological evaluation of propolis from Poland. Molecules.

[19] Ccana-Ccapatinta G.V., Mejía J.A.A., Tanimoto M.H., Groppo M., de Carvalho J.C.A.S., Bastos J.K. (2020). *Dalbergia ecastaphyllum* (L.) Taub. and *Symphonia globulifera* L.f. and botanical sources of isoflavonoids and benzophenones in Brazilian red propolis. Molecules.

[20] Tang T.X., Guo W.Y., Xu Y., Zhang S.M., Xu X.J., Wang D.M., Zhao Z.M., Zhu L.P., Yang D.P. (2014). Thin-layer chromatographic identification of chinese propolis using chemometric fingerprinting. Phytochem. Anal..

[21] Widelski J., Okińczyc P., Paluch E., Mroczek T., Szperlik J., Żuk M., Sroka Z., Sakipova Z., Chinou I., Skalicka-Woźniak K. (2022). The antimicrobial properties of poplar and aspen–poplar propolises and their active components against selected microorganisms, including *Helicobacter pylori*. Pathogens.

[22] Okińczyc P. (2017). Phytochemical Characteristics and Biological Activity of Different Kinds of Propolis and their Plant Sources [Charakterystyka Fitochemiczna Oraz Aktywność Biologiczna Różnych Rodzajów Propolisów Oraz ich Źródeł Roślinnych]. Ph.D. Thesis.

[23] Ristivojević P., Andrić F.L., Trifković J.D., Vovk I., Stanisavljević L.Z., Tešić Ž.L., Milojković-Opsenica D.M. (2014). Pattern recognition methods and multivariate image analysis in HPTLC fingerprinting of propolis extracts. J. Chemom..

[24] Saftić L., Peršurić Ž., Fornal E., Pavlešić T., Kraljević Pavelić S. (2019). Targeted and untargeted LC-MS polyphenolic profiling and chemometric analysis of propolis from different regions of Croatia. J. Pharm. Biomed. Anal..

[25] Nichitoi M.M., Josceanu A.M., Isopescu R.D., Isopencu G.O., Geana E.I., Ciucure C.T., Lavric V. (2021). Polyphenolics profile effects upon the antioxidant and antimicrobial activity of propolis extracts. Sci. Rep..

[26] Sawaya A.C.H.F., Tomazela D.M., Cunha I.B.S., Bankova V.S., Marcucci M.C., Custodio A.R., Eberlin M.N. (2004). Electrospray ionization mass spectrometry fingerprinting of propolis. Analyst.

[27] Šiler B., Skorić M., Mišić D., Kovačević B., Jelić M., Panteković A. (2014). Variability of European Black Poplar (Populus nigra L.) in the Danube Basin.

[28] Okińczyc P., Widelski J., Szperlik J., Żuk M., Mroczek T., Skalicka-Woźniak K., Sakipova Z., Widelska G., Kuś P.M. (2021). Impact of plant origin on Eurasian propolis on phenolic profile and classical antioxidant activity. Biomolecules.

[29] Popova M., Silici S., Kaftanoglu O., Bankova V. (2005). Antibacterial activity of Turkish propolis and its qualitative and quantitative chemical composition. Phytomedicine.

[30] English S., Greenaway W., Whatley F.R. (1991). Analysis of phenolics of bud exudate of *Populus euphratica* by GC-MS. Z. Naturforsch.-Sect. C J. Biosci..

[31] Isidorov V.A., Brzozowska M., Czyzewska U., Glinka L. (2008). Gas chromatographic investigation of phenylpropenoid glycerides from aspen (*Populus tremula* L.) buds. J. Chromatogr. A.

[32] Asakawa Y., Takemoto T., Wollenweber E., Aratani T. (1977). Lasiocarpin A, B and C, three novel phenolic triglycerides from *Populus lasiocarpa*. Phytochemistry.

[33] Korbik M. (2020). A review of systematics of *Populus* L. [Przegląd systematyki rodzaju *Populus* L.]. Rocz. Pol. Tow. Dendrol..

[34] Przybylski T., Białobok S., Bugała W., Hejnowicz A., Jakuszewski T., Środoń A., Obmiński Z. (1973). Nasze Drzewa Leśne: Topole Populus L..

[35] Isidorov V.A. (2007). Alchemy of Bees. Bees and Bee Products in Chemist Eyes [Alchemia pszczół. Pszczoły i Produkty Pszczele Oczami Chemika].

[36] Dimkić I., Ristivojević P., Janakiev T., Berić T., Trifković J., Milojković-Opsenica D., Stanković S. (2016). Phenolic profiles and antimicrobial activity of various plant resins as potential botanical sources of Serbian propolis. Ind. Crops Prod..

[37] Midorikawa K., Banskota A.H., Tezuka Y., Nagaoka T., Matsushige K., Message D., Huertas A.A.G., Kadota S. (2001). Liquid chromatography-mass spectrometry analysis of propolis. Phytochem. Anal..

[38] López B.G.C., Schmidt E.M., Eberlin M.N., Sawaya A.C.H.F. (2014). Phytochemical markers of different types of red propolis. Food Chem..

[39] Piccinelli A.L., Lotti C., Campone L., Cuesta-Rubio O., Campo Fernandez M., Rastrelli L. (2011). Cuban and Brazilian red propolis: Botanical origin and comparative analysis by high-performance liquid chromatography-photodiode array detection/ electrospray ionization tandem mass spectrometry. J. Agric. Food Chem..

[40] Trusheva B., Popova M., Koendhori E.B., Tsvetkova I., Naydenski C., Bankova V. (2011). Indonesian propolis: Chemical composition, biological activity and botanical origin. Nat. Prod. Res..

[41] Kumazawa S., Nakamura J., Murase M., Miyagawa M., Ahn M.R., Fukumoto S. (2008). Plant origin of Okinawan propolis: Honeybee behavior observation and phytochemical analysis. Naturwissenschaften.

[42] Banskota A.H., Tezuka Y., Prasain J.K., Matsushige K., Saiki I., Kadota S. (1998). Chemical constituents of Brazilian propolis and their cytotoxic activities. J. Nat. Prod..

[43] Salatino A., Teixeira É.W., Negri G., Message D. (2005). Origin and chemical variation of Brazilian propolis. Evid.-Based Complement. Altern. Med..

[44] Nina N., Quispe C., Jiménez-Aspee F., Theoduloz C., Feresín G.E., Lima B., Leiva E., Schmeda-Hirschmann G. (2015). Antibacterial activity, antioxidant effect and chemical composition of propolis from the Región del Maule, central Chile. Molecules.

[45] Oliveira A.V., Ferreira A.L., Nunes S., Dandlen S.A., Miguel M.D.G., Faleiro M.L. (2017). Antibacterial activity of propolis extracts from the south of Portugal. Pak. J. Pharm. Sci..

[46] Popova M.P., Graikou K., Chinou I., Bankova V.S. (2010). GC-MS profiling of diterpene compounds in mediterranean propolis from Greece. J. Agric. Food Chem..

[47] Kalogeropoulos N., Konteles S.J., Troullidou E., Mourtzinos I., Karathanos V.T. (2009). Chemical composition, antioxidant activity and antimicrobial properties of propolis extracts from Greece and Cyprus. Food Chem..

[48] Graikou K., Popova M., Gortzi O., Bankova V., Chinou I. (2016). Characterization and biological evaluation of selected Mediterranean propolis samples. Is it a new type?. LWT-Food Sci. Technol..

[49] Bankova V. (2005). Chemical diversity of propolis and the problem of standardization. J. Ethnopharmacol..

[50] Melliou E., Chinou I. (2004). Chemical analysis and antimicrobial activity of Greek propolis. Planta Med..

[51] Vardar-Ünlü G., Silici S., Ünlü M. (2008). Composition and in vitro antimicrobial activity of *Populus* buds and poplar-type propolis. World J. Microbiol. Biotechnol..

[52] Mirzoeva O.K., Grishanin R.N., Calder P.C. (1997). Antimicrobial action of propolis and some of its components: The effects on growth, membrane potential and motility of bacteria. Microbiol. Res..

[53] Scheller S., Dworniczak S., Waldemar-Klimmek K., Rajca M., Tomczyk A., Shani J. (1999). Synergism between ethanolic extract of propolis (EEP) and anti-tuberculosis drugs on growth of mycobacteria. Z. Naturforsch.-Sect. C J. Biosci..

[54] Bonvehí J.S., Gutiérrez A.L. (2012). The antimicrobial effects of propolis collected in different regions in the Basque Country (Northern Spain). World J. Microbiol. Biotechnol..

[55] Widelski J., Okińczyc P., Suśniak K., Malm A., Bozhadze A., Jokhadze M., Korona-Głowniak I. (2023). Correlation between chemical profile of Georgian propolis extracts and their activity against *Helicobacter pylori*. Molecules.

[56] Romero M., Freire J., Pastene E., García A., Aranda M., González C. (2019). Propolis polyphenolic compounds affect the viability and structure of Helicobacter pylori in vitro. Rev. Bras. Farmacogn..

[57] Okińczyc P., Paluch E., Franiczek R., Widelski J., Wojtanowski K.K., Mroczek T., Krzyżanowska B., Skalicka-Woźniak K., Sroka Z. (2020). Antimicrobial activity of Apis mellifera L. and Trigona sp. propolis from Nepal and its phytochemical analysis. Biomed. Pharmacother..

[58] Nostro A., Cellini L., Di Bartolomeo S., Di Campli E., Grande R., Cannatelli M.A., Marzio L., Alonzo V. (2005). Antibacterial effect of plant extracts against *Helicobacter pylori*. Phyther. Res..

[59] Uzel A., Sorkun K., Önçağ Ö., Çoğulu D., Gençay Ö., Sali˙h B. (2005). Chemical compositions and antimicrobial activities of four different Anatolian propolis samples. Microbiol. Res..

[60] Park Y.K., Ikegaki M. (1998). Preparation of water and ethanolic extracts of propolis and evaluation of the preparations. Biosci. Biotechnol. Biochem..

[61] Muli E.M., Maingi J.M. (2007). Antibacterial activity of *Apis mellifera* L. propolis collected in three regions of Kenya. J. Venom. Anim. Toxins incl. Trop. Dis..

[62] Abza G.B., Ahmed J.H., Paulos G., Seshathri K. (2022). In vitro analysis of the antimicrobial properties of propolis collected from Jimma, Ethiopia. J. Nat. Remedies.

[63] Fernandas A., Balestrin E.C.D., Cunha M.L.R.S. (2004). Anti Staphylococcus aureus activity of bee propolis extracts prepared with different ethanol concentrations. Rev. Cienc. Farm..

[64] Grecka K., Kuś P.M., Okińczyc P., Worobo R.W., Walkusz J., Szweda P. (2019). The anti-staphylococcal potential of ethanolic Polish propolis extracts. Molecules.

[65] Takaisi-Kikuni N.B., Schilcher H. (1994). Electron microscopic and microcalorimetric investigations of the possible mechanism of the antibacterial action of a defined propolis provenance. Planta Med..

[66] Sabuncuoglu M.Z., Kismet K., Kilicoglu S.S., Kilicoglu B., Erel S., Muratoglu S., Sunay A.E., Erdemli E., Akkus M.A. (2007). Propolis reduces bacterial translocation and intestinal villus atrophy in experimental obstructive jaundice. World J. Gastroenterol..

[67] Koru O., Toksoy F., Acikel C.H., Tunca Y.M., Baysallar M., Uskudar Guclu A., Akca E., Ozkok Tuylu A., Sorkun K., Tanyuksel M. (2007). In vitro antimicrobial activity of propolis samples from different geographical origins against certain oral pathogens. Anaerobe.

[68] Widelski J., Okińczyc P., Suśniak K., Malm A., Paluch E., Sakipov A., Zhumashova G., Ibadullayeva G., Sakipova Z., Korona-Głowniak I. (2023). Phytochemical profile and antimicrobial potential of propolis samples from Kazakhstan. Molecules.

[69] Seidel V., Peyfoon E., Watson D.G., Fearnley J. (2008). Comparative study of the antibacterial activity of propolis from different geographical and climatic zones. Phytother. Res..

[70] Drago L., Mombelli B., De Vecchi E., Fassina M.C., Tocalli L., Gismondo M.R. (2000). In vitro antimicrobial activity of propolis dry extract. J. Chemother..

[71] AL-Ani I., Zimmermann S., Reichling J., Wink M. (2018). Antimicrobial activities of European propolis collected from various geographic origins alone and in combination with antibiotics. Medicines.

[72] Okińczyc P., Widelski J., Ciochoń M., Paluch E., Bozhadze A., Jokhadze M., Mtvarelishvili G., Korona-Głowniak I., Krzyżanowska B., Kuś P.M. (2022). Phytochemical profile, plant precursors and some properties of Georgian propolis. Molecules.

[73] Ristivojević P., Dimkić I., Trifković J., Berić T., Vovk I., Milojkovič-Opsenica D., Stanković S. (2016). Antimicrobial activity of Serbian propolis evaluated by means of MIC, HPTLC, bioautography and chemometrics. PLoS ONE.

[74] Oliveira R.D., Araújo C., Almeida-Aguiar C. (2024). In vitro antimicrobial potential of Portuguese propolis extracts from Gerês against pathogenic microorganisms. Antibiotics.

[75] Dziedzic A., Kubina R., Wojtyczka R.D., Kabała-Dzik A., Tanasiewicz M., Morawiec T. (2013). The antibacterial effect of ethanol extract of polish propolis on mutans streptococci and lactobacilli isolated from saliva. Evid.-Based Complement. Altern. Med..

[76] Scheller S., Kawalski H., Oklek K., Dworniczak S., Matsuno T., Waldemar-Klimmek K., Rajca M., Shani J. (1998). Correlation between virulence of various strains of mycobacteria and their susceptibility to ethanolic extract of propolis (EEP). Z. Naturforsch.-Sect. C J. Biosci..

[77] Wojtyczka R.D., Dziedzic A., Idzik D., Kepa M., Kubina R., Kabała-Dzik A., Smoleń-Dzirba J., Stojko J., Sajewicz M., Wasik T.J. (2013). Susceptibility of *Staphylococcus aureus* clinical isolates to propolis extract alone or in combination with antimicrobial drugs. Molecules.

[78] Bouchelaghem S., Das S., Naorem R.S., Czuni L., Papp G., Kocsis M. (2022). Evaluation of total phenolic and flavonoid vontents, antibacterial and antibiofilm activities of Hungarian propolis ethanolic extract against *Staphylococcus aureus*. Molecules.

[79] Sang H., Feng K., Ju Y., Sun Y., Zhang Y., Xuan H., Wang F. (2025). Propolis exerts antibiofilm activity against methicillin-resistant Staphylococcus aureus by modulating gene expression to suppress adhesion. Microorganisms.

[80] Scazzocchio F., D’Auria F.D., Alessandrini D., Pantanella F. (2006). Multifactorial aspects of antimicrobial activity of propolis. Microbiol. Res..

[81] Dżugan M., Miłek M., Ciszkowicz E., Łyskowski A., Tomczyk M. (2025). Selected polyphenols of Polish poplar propolis as a key component shaping its antibacterial properties—In vitro and in silico approaches. Molecules.

[82] Esertaş Ü.Z., Inal N., Can Z., Kolaylı S. (2025). A promising antibacterial inhibition of propolis extracts against carbapenem-resistant bacteria isolated from clinical samples. Chem. Biodivers..

[83] Campana R., Patrone V., Tarsilla I., Franzini M., Diamantini G., Vittoria E., Baffone W. (2009). Antimicrobial activity of two propolis samples against human Campylobacter jejuni. J. Med. Food.

[84] Dinat S., Orchard A., Ncube E., Chen W., Viljoen A., van Vuuren S. (2025). South African propolis: Anti-Helicobacter pylori activity, chemistry, and toxicity. Chem. Biodivers..

[85] Navarro-Navarro M., Ruiz-Bustos P., Valencia D., Robles-Zepeda R., Ruiz-Bustos E., Virués C., Hernandez J., Domínguez Z., Velazquez C. (2013). Antibacterial activity of sonoran propolis and some of its constituents against clinically significant vibrio species. Foodborne Pathog. Dis..

[86] Belmehdi O., Bouyahya A., Jekő J., Cziáky Z., Zengin G., Sotkó G., El baaboua A., Senhaji N.S., Abrini J. (2021). Synergistic interaction between propolis extract, essential oils, and antibiotics against Staphylococcus epidermidis and methicillin resistant *Staphylococcus aureus*. Int. J. Second. Metab..

[87] Probst I.S., Sforcin J.M., Rall V.L.M., Fernandes A.A.H., Fernandes J.A. (2011). Antimicrobial activity of propolis and essential oils and synergism between these natural products. J. Venom. Anim. Toxins Incl. Trop. Dis..

[88] Okińczyc P., Szumny A., Sroka Z., Politowicz J. Headspace analyzes of volatile components of crude propolis and its extract. Proceedings of the Puzzel 2015—IV Wrocławska Konferencja Studentów Nauk Technicznych i Ścisłych.

[89] Gajger I.T., Pavlović I., Bojić M., Kosalec I., Srečec S., Vlainić T., Vlainić J. (2017). The components responsible for the antimicrobial activity of propolis from continental and mediterranean regions in Croatia. Czech. J. Food Sci..

[90] Stanciauskaite M., Marksa M., Liaudanskas M., Ivanauskas L., Ivaskiene M., Ramanauskiene K. (2021). Extracts of poplar buds (*Populus balsamifera* L., *Populus nigra* L.) and lithuanian propolis: Comparison of their composition and biological activities. Plants.

[91] Suleman T., van Vuuren S., Sandasi M., Viljoen A.M. (2015). Antimicrobial activity and chemometric modelling of South African propolis. J. Appl. Microbiol..

[92] Cushnie T.P.T., Lamb A.J. (2005). Antimicrobial activity of flavonoids. Int. J. Antimicrob. Agents.

[93] Tsuchiya H., Sato M., Miyazaki T., Fujiwara S., Tanigaki S., Ohyama M., Tanaka T., Takase I., Linuma M. (1996). Comparative study on the antibacterial activity of phytochemical flavanones against methicillin-resistant *Staphylococcus aureus*. J. Ethnopharmacol..

[94] Shamsudin N.F., Ahmed Q.U., Mahmood S., Shah S.A.A., Khatib A., Mukhtar S., Alsharif M.A., Parveen H., Zakaria Z.A. (2022). Antibacterial effects of flavonoids and their structure-activity relationship study: A comparative interpretation. Molecules.

[95] Kharsany K., Viljoen A., Leonard C., van Vuuren S. (2019). The new buzz: Investigating the antimicrobial interactions between bioactive compounds found in South African propolis. J. Ethnopharmacol..

[96] Ristivojević P., Trifković J., Andrić F., Milojković-Opsenica D. (2015). Poplar-type propolis: Chemical composition, botanical origin and biological activity. Nat. Prod. Commun..

[97] Biva I.J., Ndi C.P., Griesser H.J., Semple S.J. (2016). Antibacterial constituents of Eremophila alternifolia: An Australian aboriginal traditional medicinal plant. J. Ethnopharmacol..

[98] Du Toit K., Buthelezi S., Bodenstein J. (2009). Anti-inflammatory and antibacterial profiles of selected compounds found in south african Propolis. S. Afr. J. Sci..

[99] Boisard S., Le Ray A.M., Landreau A., Kempf M., Cassisa V., Flurin C., Richomme P. (2015). Antifungal and antibacterial metabolites from a French poplar type propolis. Evid.-Based Complement. Altern. Med..

[100] Kartal M., Yildiz S., Kaya S., Kurucu S., Topçu G. (2003). Antimicrobial activity of propolis samples from two different regions of Anatolia. J. Ethnopharmacol..

[101] Collins W., Lowen N., Blake D.J. (2019). Caffeic acid esters are effective bactericidal compounds against paenibacillus larvae by altering intracellular oxidant and antioxidant levels. Biomolecules.

[102] Maciejewicz W., Scheller S., Daniewski M. (1983). Gas chromatography—mass spectrometry investigation of propolis. Analysis of sesquiterpenes. Acta Pol. Pharm..

[103] Pellati F., Prencipe F.P., Benvenuti S. (2013). Headspace solid-phase microextraction-gas chromatography-mass spectrometry characterization of propolis volatile compounds. J. Pharm. Biomed. Anal..

[104] Loya-hernández L.P., Arzate-quintana C., Castillo-gonzález A.R., Camarillo-cisneros J., Romo-sáenz C.I., Favila-pérez M.A., Quiñonez-flores C.M. (2025). Propolis-functionalized biomaterials for wound healing: A systematic review with emphasis on polysaccharide-based platforms. Polysaccharides.

[105] Stojko M., Wolny D., Włodarczyk J. (2024). Nonwoven releasing propolis as a potential new wound healing method—A review. Molecules.

[106] Cushnie T.P.T., Lamb A.J. (2005). Detection of galangin-induced cytoplasmic membrane damage in Staphylococcus aureus by measuring potassium loss. J. Ethnopharmacol..

[107] He M., Wu T., Pan S., Xu X. (2014). Antimicrobial mechanism of flavonoids against Escherichia coli ATCC 25922 by model membrane study. Appl. Surf. Sci..

[108] Popova M., Bankova V., Butovska D., Petkov V., Nikolova-Damyanova B., Sabatini A.G., Marcazzan G.L., Bogdanov S. (2004). Validated methods for the quantification of biologically active constituents of poplar-type propolis. Phytochem. Anal..

[109] Bankova V., Popova M., Bogdanov S., Sabatini A.G. (2002). Chemical composition of European propolis: Expected and unexpected results. Z. Naturforsch.-Sect. C J. Biosci..

[110] Bankova V.S., Popov S.S., Marekov N.L. (1983). A study on flavonoids of propolis. J. Nat. Prod..

[111] Popova M., Trusheva B., Antonova D., Cutajar S., Mifsud D., Farrugia C., Tsvetkova I., Najdenski H., Bankova V. (2011). The specific chemical profile of Mediterranean propolis from Malta. Food Chem..

[112] Nanayakkara N.P.D., Burandt C.L., Jacob M.R. (2002). Flavonoids with activity against methicillin-resistant Staphylococcus aureus from Dalea scandens var. paucifolia. Planta Med..

[113] Raghukumar R., Vali L., Watson D., Fearnley J., Seidel V. (2010). Antimethicillin-resistant Staphylococcus aureus (MRSA) activity of “pacific propolis” and isolated prenylflavanones. Phyther. Res..

[114] Inui S., Hosoya T., Shimamura Y., Masuda S., Ogawa T., Kobayashi H., Shirafuji K., Moli R.T., Kozone I., Shin-Ya K. (2012). Solophenols B-D and Solomonin: New prenylated polyphenols isolated from propolis collected from the Solomon Islands and their antibacterial activity. J. Agric. Food Chem..

[115] El-Bassuony A., AbouZid S. (2010). A new prenylated flavanoid with antibacterial activity from propolis collected in Egypt. Nat. Prod. Commun..

[116] Urzúa A., Rezende M.C., Mascayano C., Vásquez L. (2008). A structure-activity study of antibacterial diterpenoids. Molecules.

[117] Smith E.C.J., Kaatz G.W., Seo S.M., Wareham N., Williamson E.M., Gibbons S. (2007). The phenolic diterpene totarol inhibits multidrug efflux pump activity in *Staphylococcus aureus*. Antimicrob. Agents Chemother..

[118] Hossion A.M.L., Zamami Y., Kandahary R.K., Tsuchiya T., Ogawa W., Iwado A., Sasaki K. (2011). Quercetin diacylglycoside analogues showing dual inhibition of DNA gyrase and topoisomerase IV as novel antibacterial agents. J. Med. Chem..

[119] Hilliard J.J., Krause H.M., Bernstein J.I., Fernandez J.A., Nguyen V., Ohemeng K.A., Barrett J.F. (1996). A comparison of active site binding of 4-quinolones and novel flavone gyrase inhibitors to DNA gyrase. Adv. Exp. Med. Biol..

[120] Mori A., Nishino C., Enoki N., Tawata S. (1987). Antibacterial activity and mode of action of plant flavonoids against *Proteus vulgaris* and *Staphylococcus aureus*. Phytochemistry.

[121] Ohemeng K.A., Schwender C.F., Fu K.P., Barrett J.F. (1993). DNA gyrase inhibitory and antibacterial activity of some flavones(1). Bioorg. Med. Chem. Lett..

[122] Plaper A., Golob M., Hafner I., Oblak M., Šolmajer T., Jerala R. (2003). Characterization of quercetin binding site on DNA gyrase. Biochem. Biophys. Res. Commun..

[123] Bernard F.X., Sablé S., Cameron B., Provost J., Desnottes J.F., Crouzet J., Blanche F. (1997). Glycosylated flavones as selective inhibitors of topoisomerase IV. Antimicrob. Agents Chemother..

[124] Cushnie T.P.T., Hamilton V.E.S., Chapman D.G., Taylor P.W., Lamb A.J. (2007). Aggregation of *Staphylococcus aureus* following treatment with the antibacterial flavonol galangin. J. Appl. Microbiol..

[125] Cui K., Lu W., Zhu L., Shen X., Huang J. (2013). Caffeic acid phenethyl ester (CAPE), an active component of propolis, inhibits *Helicobacter pylori* peptide deformylase activity. Biochem. Biophys. Res. Commun..

[126] Simuth J., Trnovsky J., Jelokova J. (1986). Inhibition of bacterial DNA-dependent RNA polymerases and restriction endonuclease by UV-absorbing components from propolis. Pharmazie.

[127] Haraguchi H., Tanimoto K., Tamura Y., Mizutani K., Kinoshita T. (1998). Mode of antibacterial action of retrochalcones from *Glycyrrhiza inflata*. Phytochemistry.

[128] Vadillo-rodr V., Fern I., Ciro P. (2024). Anti-biofilm perspectives of propolis against *Staphylococcus epidermidis* infections. Biomolecules.

[129] Wojtyczka R.D., Ma B., Idzik D., Kubina R., B. A.K., Dziedzic A., Tomasz J.W. (2013). *In vitro* antimicrobial activity of ethanolic extract of Polish propolis against biofilm forming *Staphylococcus epidermidis* strains. Evid. Based Complement. Altern. Med..

[130] Grecka K., Xiong Z.R., Chen H., Pełka K., Worobo R.W., Szweda P. (2020). Effect of ethanol extracts of propolis (EEPs) against Staphylococcal biofilm—microscopic studies. Pathogens.

[131] Queiroga M.C., Laranjo M., Andrade N., Marques M., Costa A.R., Antunes M. (2023). Antimicrobial, antibiofilm and toxicological assessment of propolis. Antibiotics.

[132] Feng K., Luan H., Sang H., Qiu W., Yang R., Cheng J., Feng W. (2026). Caffeic acid phenethyl ester suppresses adhesion to mediate its antibiofilm activity against methicillin-resistant *Staphylococcus aureus*. Microorganisms.

[133] Li T., Yuan J., Gao S., Tang Y., Xuan H. (2025). Propolis-derived components act against *Klebsiella pneumoniae* by inhibiting growth and disrupting biofilms. J. Appl. Microbiol..

[134] De Marco S., Piccioni M., Pagiotti R., Pietrella D. (2017). Antibiofilm and antioxidant activity of propolis and bud poplar resins versus *Pseudomonas aeruginosa*. Evid. Based Complement Altern. Med..

[135] Meto A., Colombari B., Meto A., Boaretto G., Pinetti D., Marchetti L., Benvenuti S., Pellati F., Blasi E. (2020). Propolis effects Pseudomonas aeruginosa growth, biofilm formation, eDNA release and phenazine production: Potential involvement of polyphenols. Microorganisms.

[136] Yuan J., Yuan W., Guo Y., Wu Q., Wang F., Xuan H. (2022). Anti-biofilm activities of Chinese poplar propolis essential oil against *Streptococcus mutans*. Nutrients.

[137] Bulman Z., Le P., Hudson A.O., Savka M.A. (2011). A novel property of propolis (bee glue): Anti-pathogenic activity by inhibition of N-acyl-homoserine lactone mediated signaling in bacteria. J. Ethnopharmacol..

[138] Hadjab W., Zellagui A., Mokrani M., Öztürk M., Ceylan Ö. (2024). Pharmacological potential effects of Algerian propolis against oxidative stress, multidrug-resistant pathogens biofilm and quorum-sensing. Turk. J. Pharm. Sci..

[139] Ong T.H., Chitra E., Ramamurthy S., Siddalingam P., Yuen K.H., Ambu S.P., Davamani F. (2017). Chitosan-propolis nanoparticle formulation demonstrates anti-bacterial activity against *Enterococcus faecalis* biofilms. PLoS ONE.

[140] Kędzia B., Hołderna-Kędzie E. (2019). Immunostymulujące działanie propolisu [Immunostimulatory activity of propolis]. Post. Fitoter..

[141] Celik S., Gorur S., Aslantas O., Erdogan S., Ocak S., Hakverdi S. (2007). Caffeic acid phenethyl ester suppresses oxidative stress in *Escherichia coli*-induced pyelonephritis in rats. Mol. Cell. Biochem..

[142] Governa P., Cusi M.G., Borgonetti V., Sforcin M., Terrosi C., Baini G., Miraldi E., Biagi M. (2022). Beyond the biological effect of a chemically characterized poplar propolis: Antibacterial and antiviral activity and comparison with flurbiprofen in cytokines release by LPS-stimulated human mononuclear cells. Biomedicines.

[143] Salama S.A., Essam D., Tagyan A.I., Farghali A.A., Khalil E.M., Abdelaleim Y.F., Hozzein W.N., Mubarak M., Nasr F.A., Eweis A.A. (2024). Novel composite of nano zinc oxide and nano propolis as antibiotic for antibiotic-resistant bacteria: A promising approach. Sci. Rep..

[144] Orsi R.O., Fernandes A., Bankova V., Sforcin J.M. (2012). The effects of Brazilian and Bulgarian propolis in vitro against *Salmonella* Typhi and their synergism with antibiotics acting on the ribosome. Nat. Prod. Res..

[145] Orsi R.D.O., Sforcin J.M., Cunha Funari S.R., Fernandes A., Bankova V. (2006). Synergistic effect of propolis and antibiotics on the *Salmonella* Typhi. Braz. J. Microbiol..

[146] Meresta T. (1997). Long-lasting action of propolis extracts on *Staphylococcus aureus* [article in Polish: Długotrwale oddzialywanie ekstraktów propolisu na Staphylococcus aureus]. Med. Weter..

[147] Garedew A., Schmolz E., Lamprecht I. (2004). Microbiological and calorimetric investigations on the antimicrobial actions of different propolis extracts: An in vitro approach. Thermochim. Acta.

[148] Savoia D. (2012). Plant-derived antimicrobial compounds: Alternatives to antibiotics. Future Microbiol..

[149] Stepanovi S., Daki I., Milena Š. (2003). In vitro antimicrobial activity of propolis and synergism between propolis and antimicrobial drugs. Microbiol. Res..

[150] Rana A., Malik A., Chander R. (2025). Antibacterial properties of propolis: A comprehensive review. Curr. Microbiol..

[151] Krol W., Scheller S., Shani J., Pietsz G., Czuba Z. (1993). Synergistic effect of ethanolic extract of propolis and antibiotics on the growth of *Staphylococcus aureus*. Arzneim.-Forsch./Drug Res..

[152] Orsi R.O., Fernandes A.J., Bankova V., Sforcin J.M. (2012). Antibacterial effects of Brazilian and Bulgarian propolis and synergistic effects with antibiotics acting on the bacterial DNA and folic acid. Nat. Prod. Res..

[153] Nostro A., Cellini L., Di Bartolomeo S., Cannatelli M.A., Di Campli E., Procopio F., Grande R., Marzio L., Alonzo V. (2006). Effects of combining extracts (from propolis or *Zingiber officinale*) with clarithromycin on *Helicobacter pylori*. Phyther. Res..

[154] Speciale A., Costanzo R., Puglisi S., Musumeci R., Catania M.R., Caccamo F., Iauk L. (2006). Antibacterial activity of propolis and its active principles alone and in combination with macrolides, beta-lactams and fluoroquinolones against microorganisms responsible for respiratory infections. J. Chemother..

[155] Onlen Y., Tamer C., Oksuz H., Duran N., Altug M.E., Yakan S. (2007). Comparative trial of different anti-bacterial combinations with propolis and ciprofloxacin on *Pseudomonas keratitis* in rabbits. Microbiol. Res..

[156] Onlen Y., Duran N., Atik E., Savas L., Altug E., Yakan S., Aslantas O. (2007). Antibacterial activity of propolis against MRSA and synergism with topical mupirocin. J. Altern. Complement. Med..

[157] Oner M., Kafadar I., Guney A., Halici M., Deniz K., Turk Y., Argun M. (2011). Effect of intraarticular propolis in an experimental septic arthritis model. J. Pediatr. Orthop. B.

[158] Enis Yonar M., Mişe Yonar S., Silici S. (2011). Protective effect of propolis against oxidative stress and immunosuppression induced by oxytetracycline in rainbow trout (*Oncorhynchus mykiss*, W.). Fish Shellfish Immunol..

[159] Tatli Seven P., Seven I., Gul Baykalir B., Iflazoglu Mutlu S., Salem A.Z.M. (2018). Nanotechnology and nano-propolis in animal production and health: An overview. Ital. J. Anim. Sci..

[160] Murtaza H., Rafique A., Iqbal S., Irfan S., Nazir M.S., Ijaz M., Hussain F., Hassan S.U. (2026). MXene-Based MnZnO nanocomposites for enhanced antibacterial activity and cutaneous wound healing. Appl. Biochem. Biotechnol..

[161] Xiao S., Sun G., Huang S., Lin C., Li Y. (2024). Nanoarchitectonics-based materials as a promising strategy in the treatment of endodontic infections. Pharmaceutics.

[162] Razavi R., Molaei R., Alipashaeihalabi A., Mirani A., Alizadeh N., Abbaszadeh M., Moradi M. (2026). Propolis–carboxymethyl cellulose Janus nanoparticles as multifunctional bioactive additives in stirred yogurt. Sci. Rep..

[163] Erdoğan E., Özdem B., Çimentepe Ö.Ö., Tekedereli I., Yıldırım I. (2026). Biochemical and cellular effects of propolis and silver nanoparticle combination: Apoptotic signaling and antibiofilm activity. Mol. Biol. Rep..

[164] Xu V.W., Nizami M.Z.I., Yin I.X., Lung C.Y.K., Yu O.Y., Chu C.H. (2022). Caries management with Non-metallic nanomaterials: A systematic review. Int. J. Nanomed..

[165] Ong T.H., Chitra E., Ramamurthy S., Chong C., Ling S., Ambu S.P., Id F.D. (2019). Cationic chitosan-propolis nanoparticles alter the zeta potential of *S. epidermidis*, inhibit biofilm formation by modulating gene expression and exhibit synergism with antibiotics. PLoS ONE.

[166] Kędzia B., Hołderna-Kędzia E. (2022). Apitherapy. Treatment with Honey and Others Bee Product [Apiterapia. Leczenie Miodem i Innymi Produktami Pszczelimi.].

[167] Kędzia B., Hołderna-Kędzia E. (2011). Propolis in Internal Diseases [Propolis w Chorobach Wewnętrznych].

[168] Mehri A., Pourmoslemi S., Khalilian A., Safari M., Ayubi E. (2026). Enhanced H. pylori treatment using a β-cyclodextrin–propolis complex: A randomized clinical trial. BioMed Res. Int..

[169] Khabazian A., Mirhashemi F., Sadeghi F. (2025). Investigating the effect of propolis-containing chewing gum in comparison with propolis- containing mouthwash on reducing gingival inflammation in patients with gingivitis. BMC Oral Health.

[170] Gunjal S., Gowda D., Pateel S. (2024). Comparative effectiveness of Propolis with chlorhexidine mouthwash on gingivitis—a randomized controlled clinical study. BMC Complement. Med. Ther..

[171] Bapat S., Nagarajappa R. (2021). Effect of propolis mouth rinse on oral microorganisms—A randomized controlled trial. Clin. Oral Investig..

[172] Morawiec T., Olek M., Mertas A., Machorowska-pieni A. (2021). Advantages of using toothpaste containing propolis and plant oils for gingivitis prevention and oral cavity hygiene in cleft lip/palate patients. Biomed. Pharmacother..

[173] Bretz W.A., Paulino N., Nör J.E., Moreira A. (2014). The effectiveness of propolis on gingivitis: A randomized controlled trial. J. Altern. Complement. Med..

[174] Peycheva S., Apostolova E., Gardjeva P., Peychev Z., Kokova V., Angelov A., Slavov A., Murdjeva M. (2019). Effect of Bulgarian propolis on the oral microflora in adolescents with plaque-induced gingivitis. Rev. Bras. Farmacogn..

[175] RojaRamya K.S., Vinay C., Uloopi K.S., Chandrasekhar R. (2020). In vivo evaluation of zinc oxide-propolis mixture as root canal filling material in the primary molars: A 24-month follow-up randomized controlled trial. J. Indian Soc. Pedod. Prev. Dent..

[176] Atanasovska Stojanovska A., Popovska M., Muratovska I., Mitic K., Stefanovska E., Radojkova Nikolovska V. (2014). Therapeutic effect of proaftol in treatment of recurrent aphthous stomatitis. Pril (Makedon. Akad. Nauk. Umet. Oddelenie Med. Nauk.).

[177] Samet N., Laurent C., Susarla S.M., Samet-Rubinsteen N. (2007). The effect of bee propolis on recurrent aphthous stomatitis: A pilot study. Clin. Oral Investig..

[178] Boeri L., De Lorenzis E., Lucignani G., Turetti M., Silvani C., Zanetti S.P., Longo F., Albo G., Salonia A., Montanari E. (2024). Oral preparation of hyaluronic acid, chondroitin sulfate, N-acetylglucosamine, and vitamin C improves sexual and urinary symptoms in participants with recurrent urinary tract infections: A randomized crossover trial. J. Sex. Med..

[179] Shekari M., Hadi A., Mahmood H., Daabo A., Hjazi A., Rafie N. (2023). Propolis as an adjunctive therapy for treatment of uncomplicated cystitis in women: A randomized double-blind. Psychother. Res..

[180] Bruyère F., Azzouzi A.R., Lavigne J.-P., Droupy S., Coloby P., Game X., Karsenty G., Issartel B., Ruffion A., Misrai V. (2019). A multicenter, randomized, placebo-controlled study evaluating the efficacy of a combination of propolis and cranberry (*Vaccinium macrocarpon*) (DUAB^®^) in preventing low urinary tract infection recurrence in women complaining of recurrent cystitis. Urol. Int..

[181] De Leo V., Cappelli V., Massaro M.G., Tosti C., Morgante G. (2017). Evaluation of the effects of a natural dietary supplement with cranberry, Noxamicina^®^ and D-mannose in recurrent urinary infections in perimenopausal women. Minerva Ginecol..

[182] Imhof M., Lipovac M., Kurz C., Barta J., Verhoeven H.C., Huber J.C. (2005). Propolis solution for the treatment of chronic vaginitis. Int. J. Gynaecol. Obstet. Off. Organ. Int. Fed. Gynaecol. Obstet..

[183] Esposito C., Ugo E., Bocchino B., Avino M.D., Caruso G., Riccardo A., Sacchi R., Galeotti F., Carlo G., Zaccaria V. (2021). A standardized polyphenol mixture extracted from poplar-type propolis for remission of symptoms of uncomplicated upper respiratory tract infection clinical trial. Phytomedicine.

[184] Marchisio P., Esposito S., Bianchini S., Desantis C., Galeone C., Nazzari E., Pignataro L., Principi N. (2010). Effectiveness of a propolis and zinc solution in preventing acute otitis media in children with a history of recurrent acute otitis media. Int. J. Immunopathol. Pharmacol..

[185] Henshaw F.R., Bolton T., Nube V., Hood A., Veldhoen D., Pfrunder L., McKew G.L., Macleod C., McLennan S.V., Twigg S.M. (2014). Topical application of the bee hive protectant propolis is well tolerated and improves human diabetic foot ulcer healing in a prospective feasibility study. J. Diabetes Complicat..

[186] Kucharzewski M., Kózka M., Urbanek T. (2013). Topical treatment of nonhealing venous leg ulcer with propolis ointment. Evid. Based Complement Altern. Med..

[187] Mbarik M., Poirier S.J., Doiron J., Selka A., Barnett D.A., Cormier M., Touaibia M., Surette M.E. (2019). Phenolic acid phenethylesters and their corresponding ketones: Inhibition of 5-lipoxygenase and stability in human blood and HepaRG cells. Pharmacol. Res. Perspect..

[188] Curti V., Zaccaria V., Jorel A., Sokeng T., Dacrema M., Masiello I., Mascaro A., Antona G.D., Daglia M. (2019). Bioavailability and in vivo antioxidant activity of a standardized polyphenol mixture extracted from brown propolis. Int. J. Mol. Sci..

[189] Gao S., Siddiqui N., Etim I., Du T., Zhang Y., Liang D. (2021). Developing nutritional component chrysin as a therapeutic agent: Bioavailability and pharmacokinetics consideration, and ADME mechanisms. Biomed. Pharmacother..

[190] Pino A., Salinas-camargo J.C., Chung L.H., Alcaraz P.E., Canela N., Cases J. (2026). Nutrikinetics and bioavailability of Promunel^®^, a standardized poplar-type propolis phenolic extract: A double-blinded, placebo-controlled, cross-over, randomized trial. Food Funct..

